# Supplementary material for: Perovskite solar cells with enhanced thermal fatigue resistance under extreme temperature cycling
Source: Nat Commun. 2026 Mar 9;17:3669. doi: 10.1038/s41467-026-70293-7 (PMC13100044; doi:10.1038/s41467-026-70293-7)
Supplement: Supplementary file 1 — Supplementary Information [file 41467_2026_70293_MOESM1_ESM.pdf]

Supplementary Information for

Perovskite Solar Cells with Enhanced Thermal Fatigue Resistance under Extreme Temperature Cycling

Cem Yilmaz[1, +], Ali Buyruk[1, +], Yating Shi[2, +], Sergej Levashov[3], Xiaole Li[4], Rik Hooijer[1], Jian Huang[1], Hao Zhu[1], Oliver Fischer[5,6], Martin C. Schubert[5,6], Caner Deger[7], Ilhan Yavuz[7], Esma Ugur[1], Gilles Lubineau[4], Johanna Eichhorn[3], Fei Zhang[2,8,9*], Erkan Aydin[1*]

[1]*Department of Chemistry, Ludwig-Maximilians-Universität München (LMU), Butenandtstraße 11 (E), 81377 Munich, Germany*

[2]*School of Chemical Engineering and Technology, Tianjin University, Tianjin 300072, China*

[3] *Physics Department, School of Natural Sciences, Technical University of Munich, Am Coulombwall 4, 85748 Garching, Germany*

[4]*Mechanics of Composites for Energy and Mobility Lab, King Abdullah University of Science and Technology (KAUST), Thuwal 23955-6900, Saudi Arabia*

[5]*Fraunhofer Institute for Solar Energy Systems ISE, Heidenhofstr. 2, Freiburg, Germany*

[6]*Chair of Photovoltaic Energy Conversion, Department of Sustainable Systems Engineering INATECH, University of Freiburg, Emmy-Noether-Str. 2, Freiburg, Germany*

[7]*Department of Physics, Marmara University, Ziverbey 34722, Türkiye*

[8]*Collaborative Innovation Center of Chemical Science and Engineering (Tianjin), Tianjin 300072, China*

[9]*Haihe Laboratory of Sustainable Chemical Transformations, Tianjin 300192, China*

[+] These authors contributed equally to this work.

[*]Corresponding author: erkan.aydin@cup.uni-muenchen.de, fei_zhang@tju.edu.cn

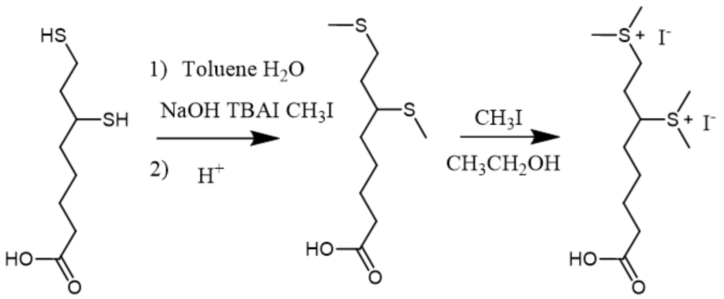

(1) (2) (3)

Supplementary Figure 1: Synthetic route for the preparation of DMSLA.

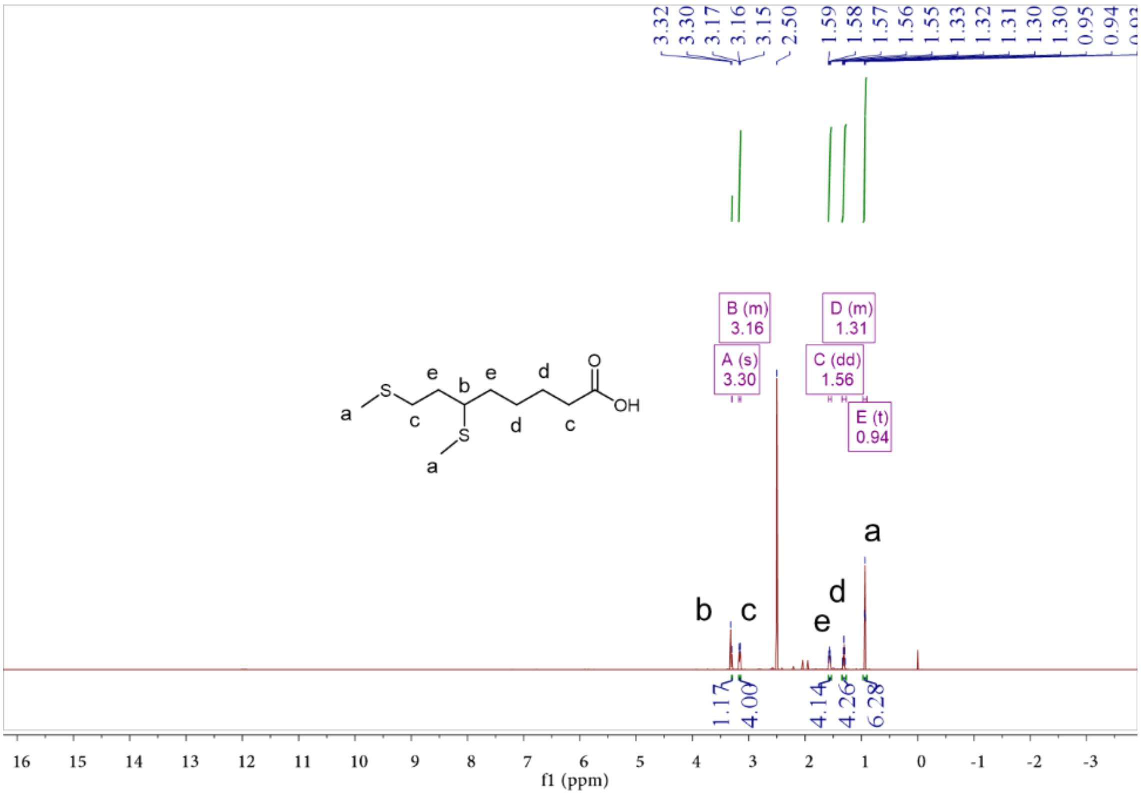

Supplementary Figure 2: ^{1}H NMR of 6,8-bis(methylthio)octanoic acid. ^{1}H NMR (800 MHz, DMSO) δ 3.30 (s, 1H), 3.18 – 3.14 (m, 4H), 1.56 (dd, J = 15.9, 8.1 Hz, 4H), 1.34 – 1.27 (m, 4H), 0.94 (t, J = 7.4 Hz, 6H).

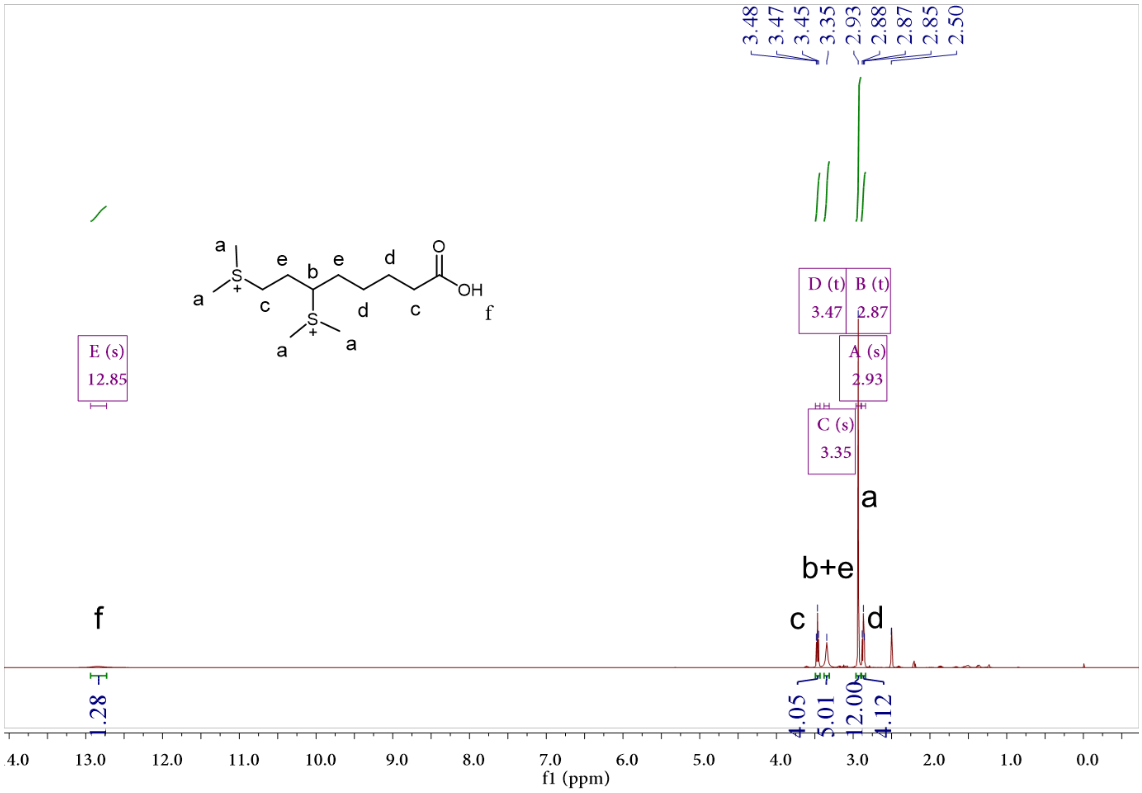

Supplementary Figure 3: ^{1}H NMR of 1,3-Bis(dimethylsulfonio)heptane-7-carboxylic acid diiodide (DMSLA). ^{1}H NMR (500 MHz, DMSO) δ 12.85 (s, 1H), 3.47 (t, J = 7.2 Hz, 4H), 3.35 (s, 5H), 2.93 (s, 12H), 2.87 (t, J = 7.2 Hz, 4H).

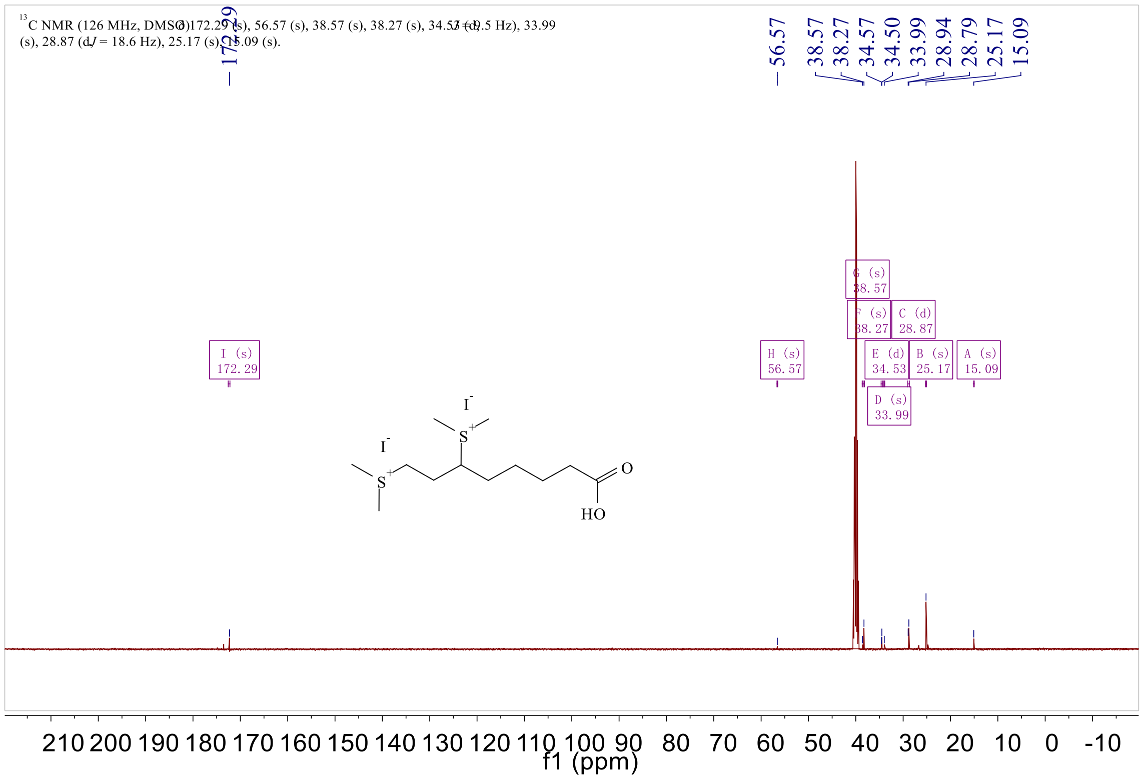

Supplementary Figure 4: ^{13}C NMR of 1,3-Bis(dimethylsulfonio)heptane-7-carboxylic acid diiodide (DMSLA). ^{13}C NMR (126 MHz, DMSO) δ 172.29 (s), 56.57 (s), 38.57 (s), 38.27 (s), 34.53 (d, J = 9.5 Hz), 33.99 (s), 28.87 (d, J = 18.6 Hz), 25.17 (s), 15.09 (s).

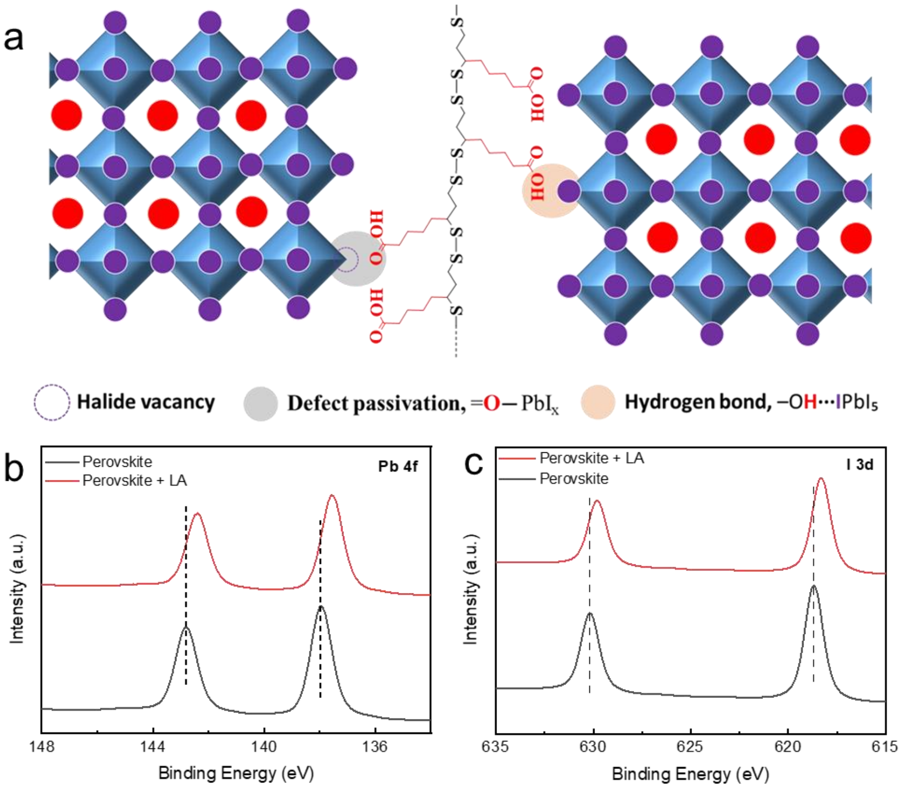

Supplementary Figure 5: a) Schematic illustration of poly(LA) formation at the perovskite grain boundaries during the annealing process. b-c) XPS spectra of Pb 4f and I 3d core energy levels in neat perovskite film (control, perovskite) and LA bulk-treated film (target, perovskite+LA).

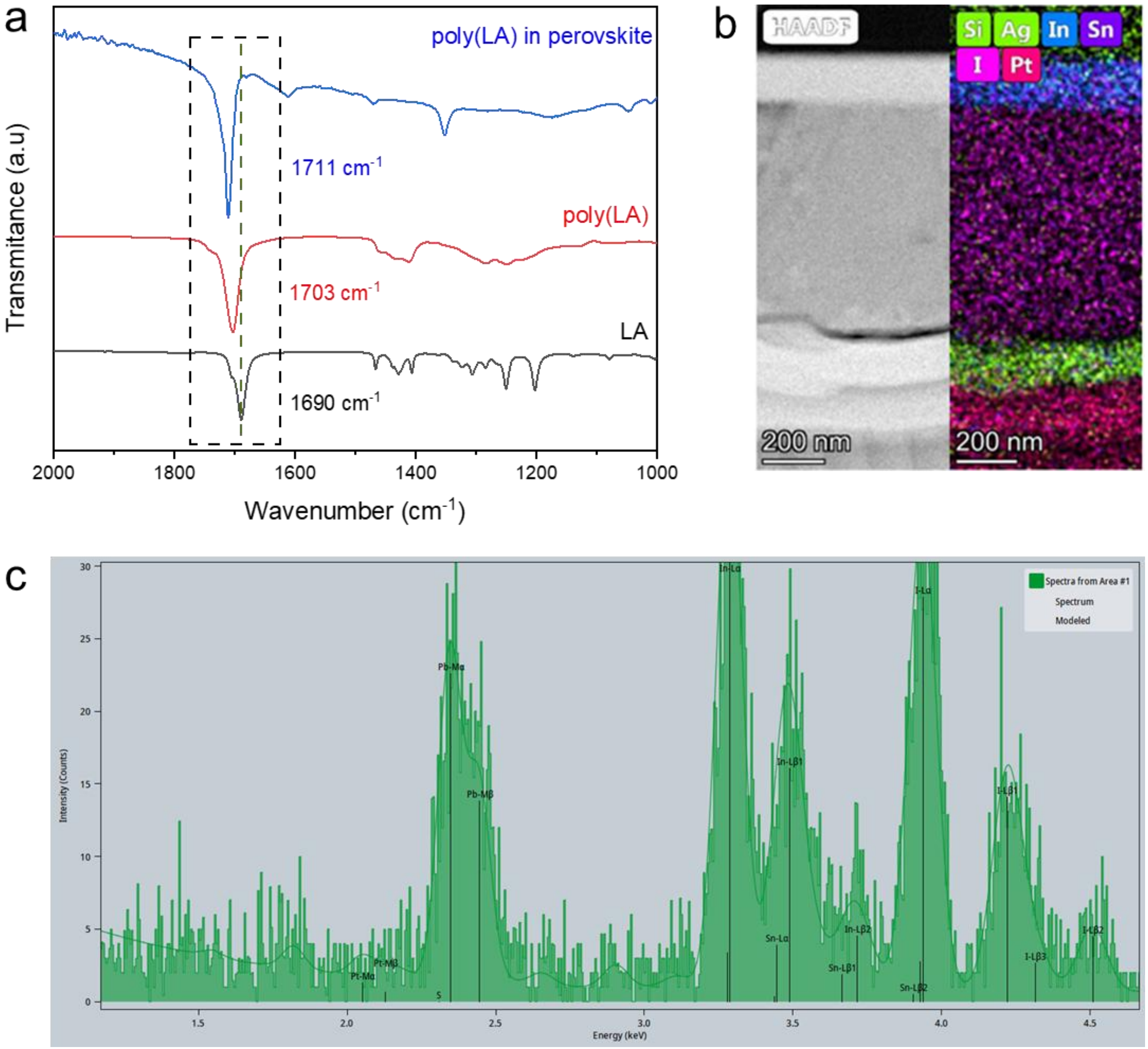

Supplementary Figure 6: a) FTIR spectra of LA, poly(LA), and poly(LA) in perovskite bulk structure. b) The high-angle annular dark-field scanning transmission electron microscopy (HAADF-STEM) image and the corresponding energy-dispersive X-ray spectroscopy (EDX) elemental mapping of the perovskite polycrystalline structure containing poly(LA). c) EDX energy spectrum obtained from the perovskite sample incorporating poly(LA) within the bulk structure. In **Supplementary Figure 6a** shown in blue, the –COOH stretching vibration in the FTIR spectrum of poly(LA) within the perovskite bulk structure is masked by the 1711 cm^{-1} antisymmetric C–N stretching of the FA^{+} cation[1], the main component of the perovskite structure. For the TEM analysis, a full device sample was prepared and examined. However, as shown in **Supplementary Figure 6c**, the EDX signals of S and Pb, observed in the range of 2.3–2.5 keV range, overlap significantly, masking the observation of sulphur atoms in poly(LA).

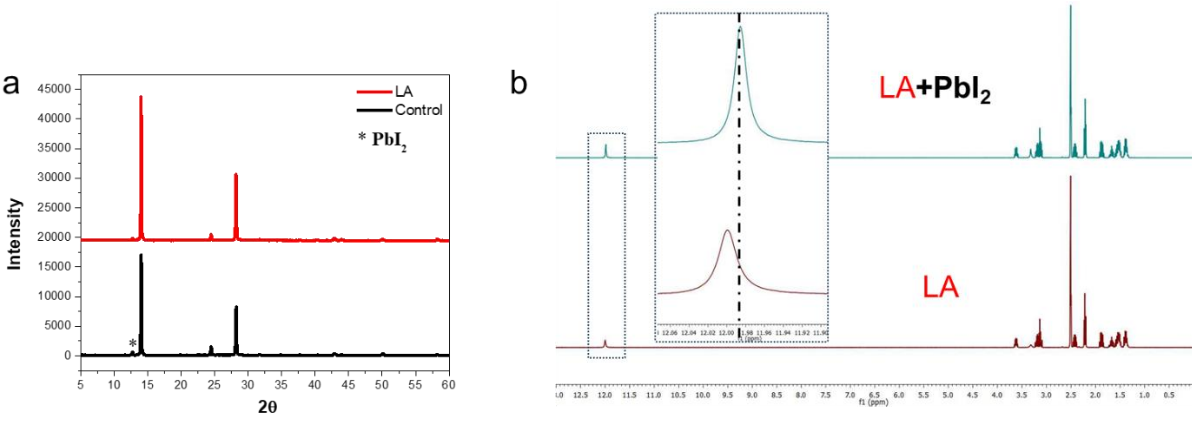

Supplementary Figure 7: a) XRD patterns of control (perovskite) and LA (perovskite + LA), the excess PbI_2 signal is marked with an asterisk (*). b) ^{1}H-NMR spectra of LA, its mixture with PbI_2.

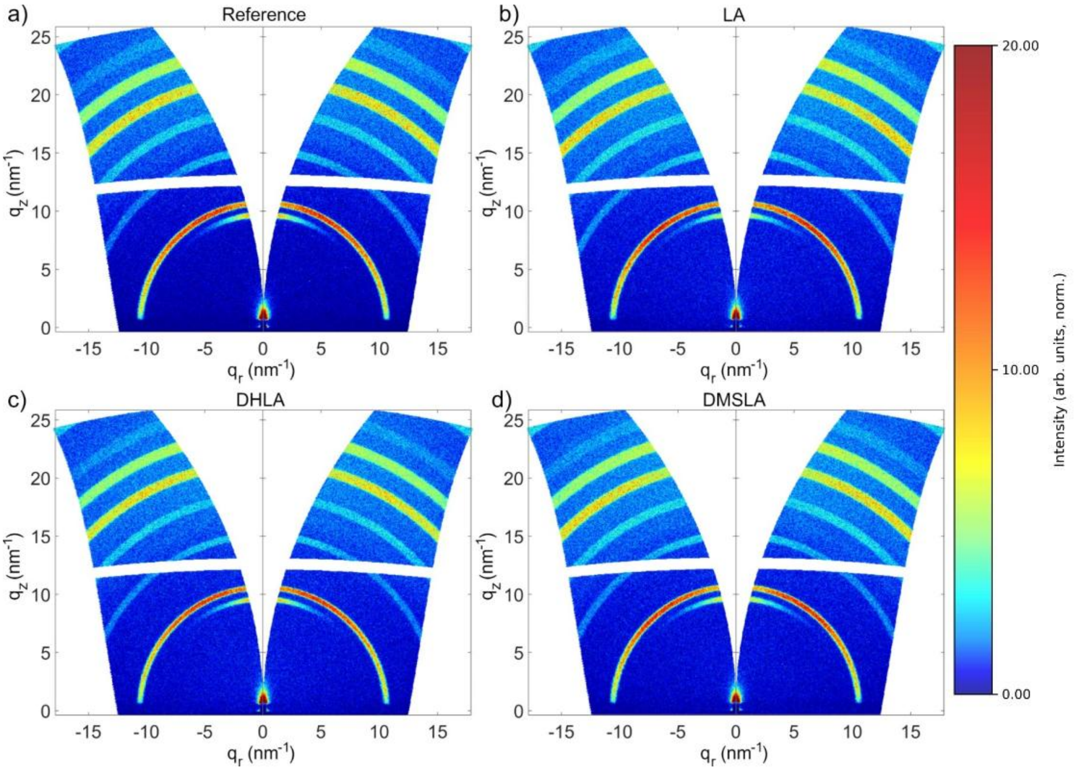

Supplementary Figure 8: GIWAXS results of perovskite films. The control group corresponds to perovskite films without any LA or its derivatives, whereas the other samples include LA and its derivatives at the perovskite–HTL interface, along with a fixed amount of LA incorporated within the bulk structure.

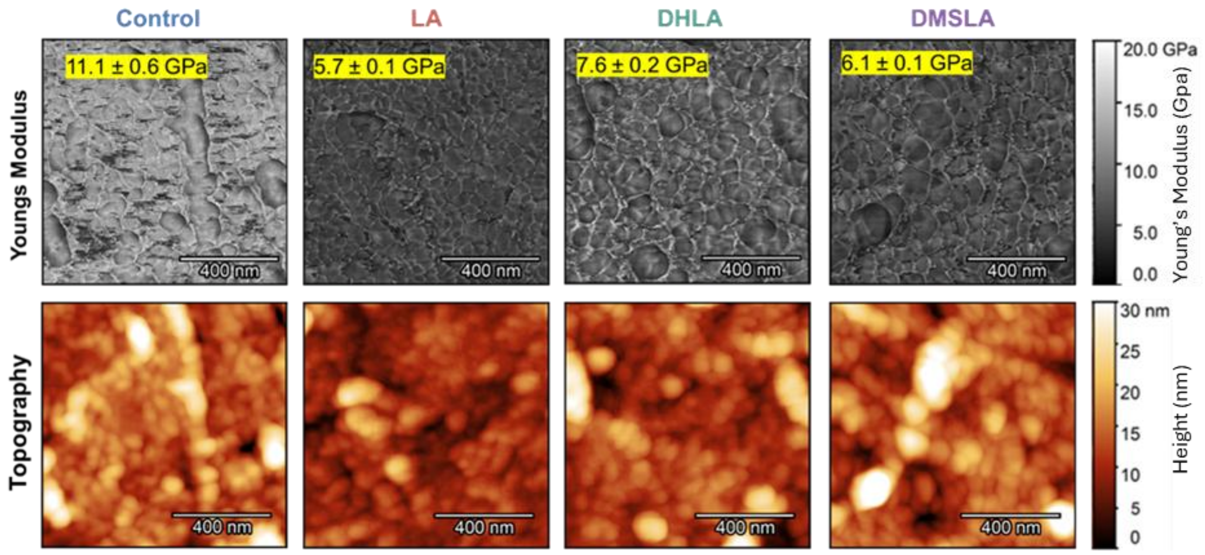

Supplementary Figure 9: Peak Force QNM (Quantitative Nanomechanical Mapping) images from the bottom contacts of control and target (LA, DHLA, DMSLA) samples. The upper row shows Young's modulus maps, and the lower row shows corresponding topographical maps. The yellow boxes at the top-left corners of the images show the average Young's modulus and standard deviation for each image, obtained alongside the corresponding topographical imaging.

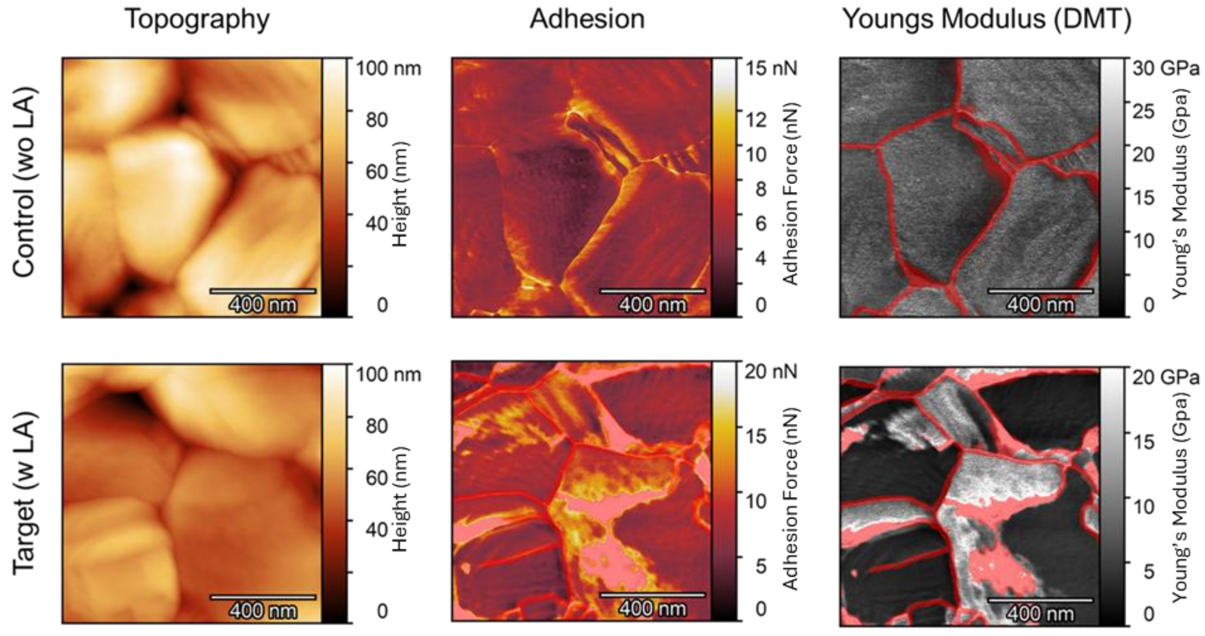

Sample (Top Contact)	YM* (grain, dark spot) [GPa]	YM* (grain, bright spot) [GPa]	YM* (boundary) [Gpa]	Adhesion (grain) [nN]	Adhesion (boundary) [nN]
Control (wo LA)	9.6 ± 1.5	-	8.5 ± 2.3	4.7 ± 0.4	7.4 ± 0.8
Target (w LA)	3.3 ± 0.5	8.9 ± 1.6	18 ± 5.7	7.7 ± 0.8	15 ± 1.3

Supplementary Figure 10: Peak Force QNM images from the top of control and target (LA) samples. From left to right: topography, adhesion-force maps, and Young's modulus maps. Red areas in the top contact represent the mask, which distinguishes between grains and grain

boundaries where the tip apex curvature can be bigger than the grain boundary depth and width, which could lead to overestimation of the mechanical properties, in addition to the enhanced properties due to the LA. The pronounced mechanical contrast and localized stiffness enhancement along the grain boundaries indicate that LA or its polymerized form likely remains within these interfacial regions after film formation, contributing to improved boundary adhesion. We note that on certain grain facets, a distinct region with lower stiffness is observed, which we attribute to LA molecules bonded to the perovskite being exposed at the surface. In contrast, the brighter areas correspond to unmodified perovskite, with stiffness values matching those of the unprocessed sample.

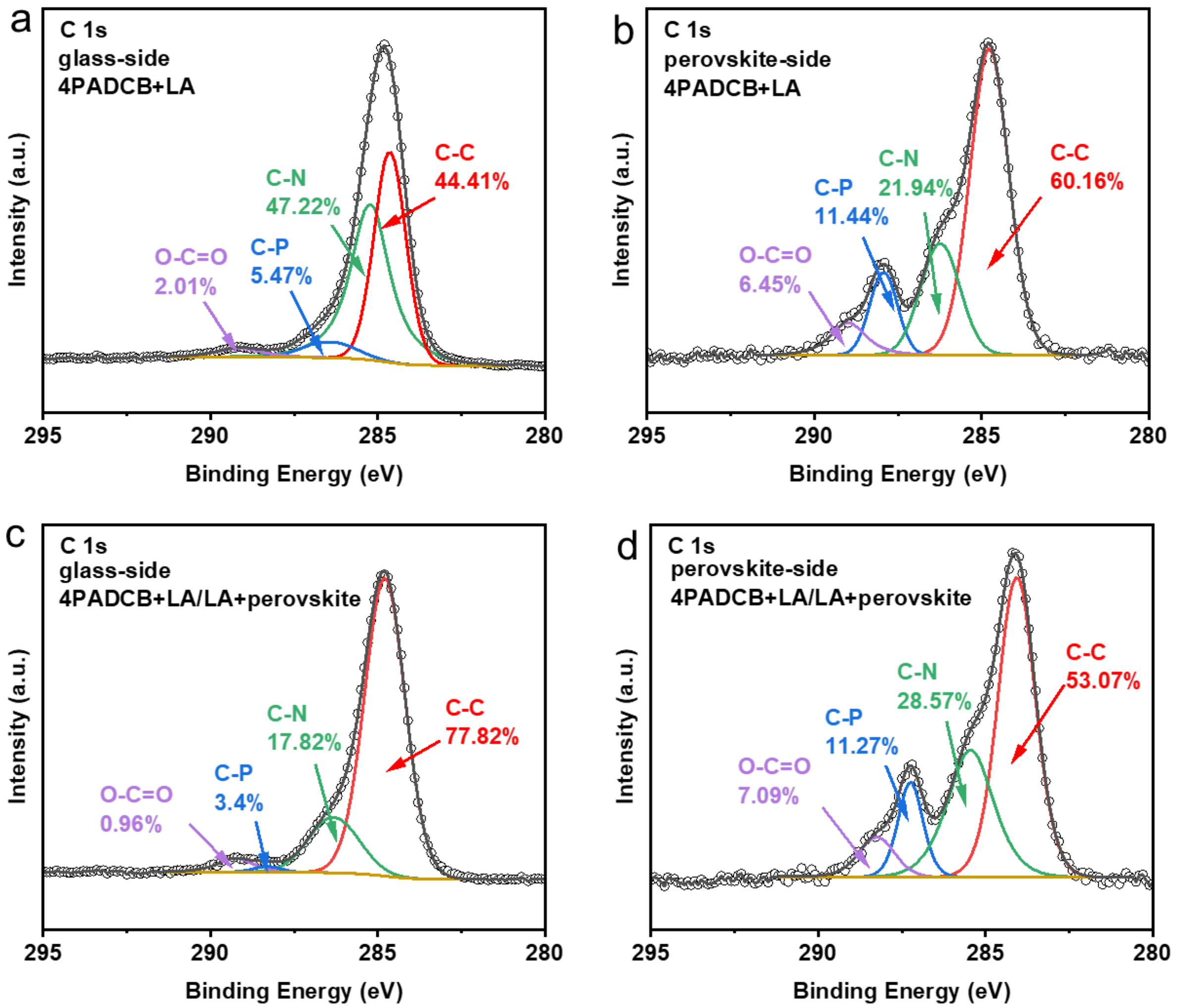

Supplementary Figure 11: XPS spectra of the C 1s core level in the pristine SAM (control) and SAM films from ITO and perovskite side incorporating LA analogues (target samples).

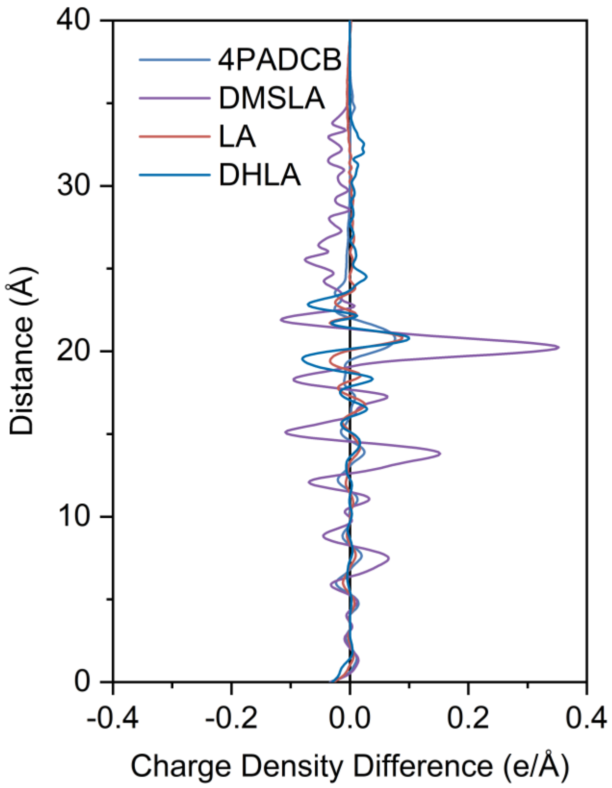

Supplementary Figure 12: Planar-averaged charge density difference (CDD) plots for 4PADCB, LA, and its derivatives at the perovskite/SAM contact.

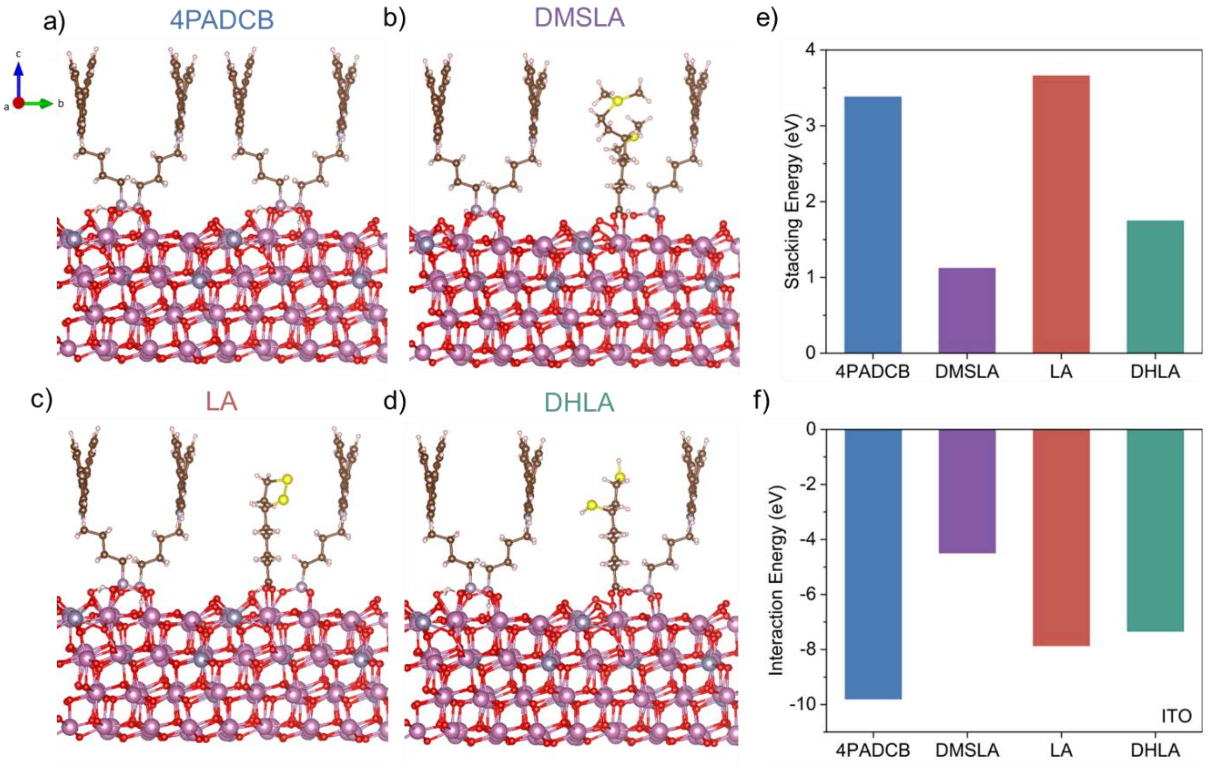

Supplementary Figure 13: DFT calculations on SAM and its blended forms with ITO interaction. While 4PADCB alone shows strong binding to ITO (–10 eV), it has a high stacking energy (+3.5 eV), indicating poor layer formation. Adding DMSLA significantly reduces the stacking energy (+0.7 eV), promoting better SAM formation and stability. DHLA also lowers the stacking energy more than pure 4PADCB, but less effectively than DMSLA, suggesting that DMSLA is the most beneficial additive for achieving a uniform and stable monolayer.

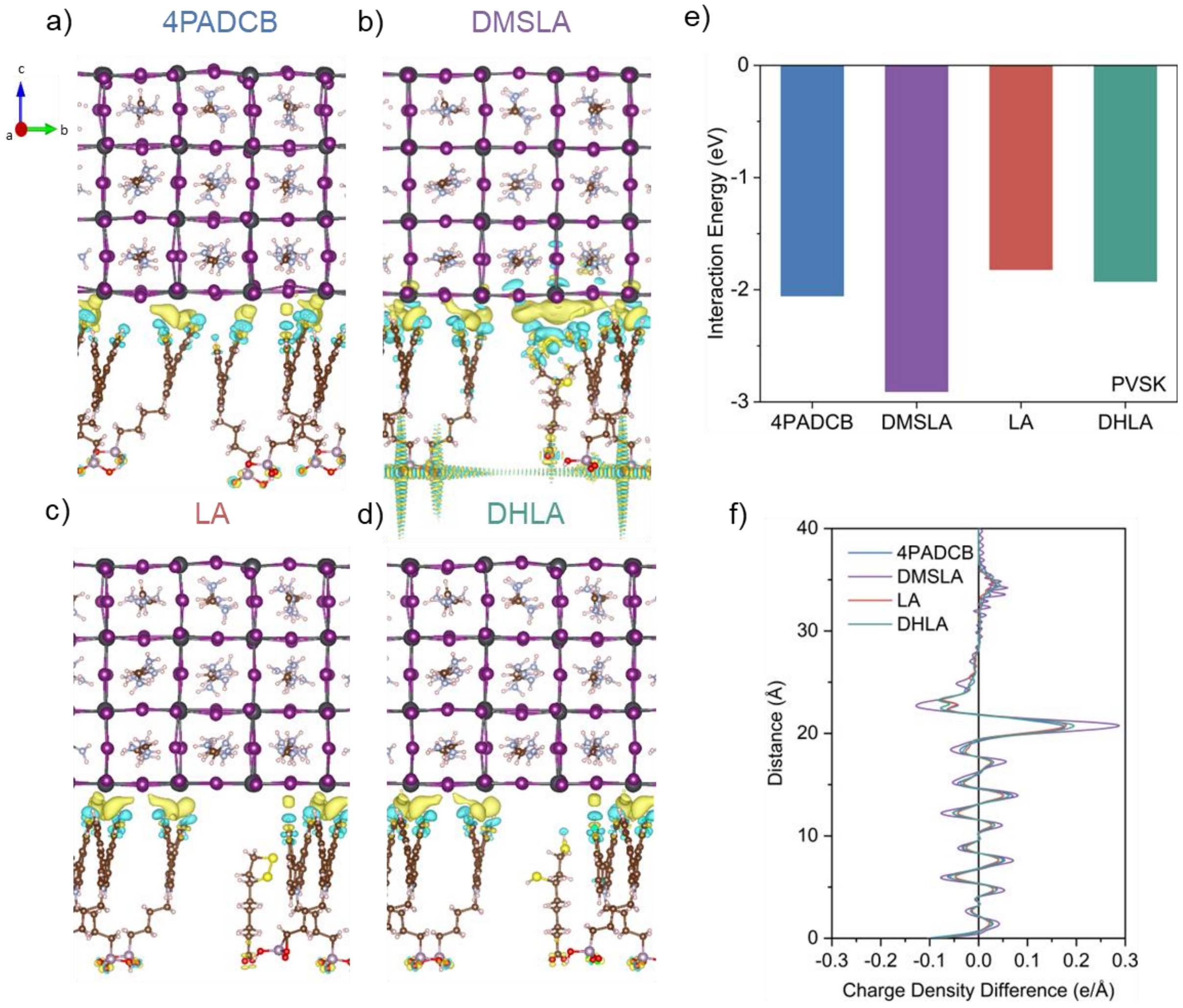

Supplementary Figure 14: DFT calculations on SAM and its blended forms with perovskite interaction. Here is a comparative analysis of SAMs—4PADCB, DMSLA, LA, and DHLA—on a perovskite (PVSK) surface. The bar chart indicates that the DMSLA additive exhibits the strongest interaction energy. At the same time, the charge density difference profiles and isosurfaces reveal significant charge transfer at the interface, as illustrated in **Supplementary Figure 14f**. Notably, DMSLA also displays enhanced charge transfer characteristics; however, the difference is slight.

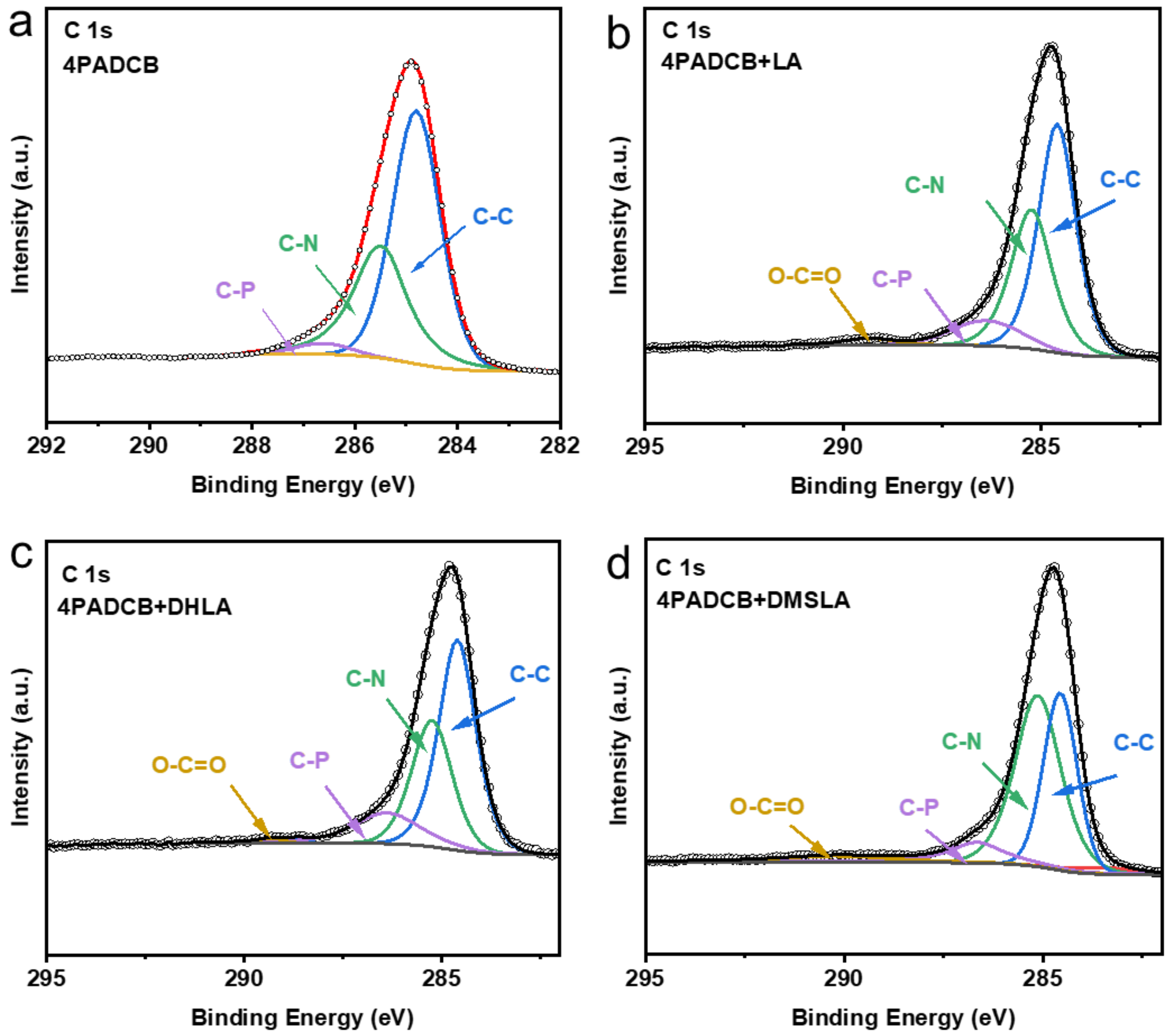

Supplementary Figure 15: XPS spectra of the C 1s core level in the pristine SAM (control) and SAM films incorporating LA analogues (target samples).

a)

Substrates	In 3d area	C1s area	Carbon atom number	Coverage factor	Molecule density
ITO/4PADCB	1023668.75	247414.82	24	1×10^{-2}	2.84×10^{13}
ITO/4PADCB+LA	1450618.78	220328.86	26	5.8×10^{-3}	3.22×10^{13}
ITO/4PADCB+DHLA	1410166.63	212380.19	26	5.8×10^{-3}	3.50×10^{13}
ITO/4PADCB+DMSLA	1431155.23	211761.37	27	5.5×10^{-3}	3.75×10^{13}

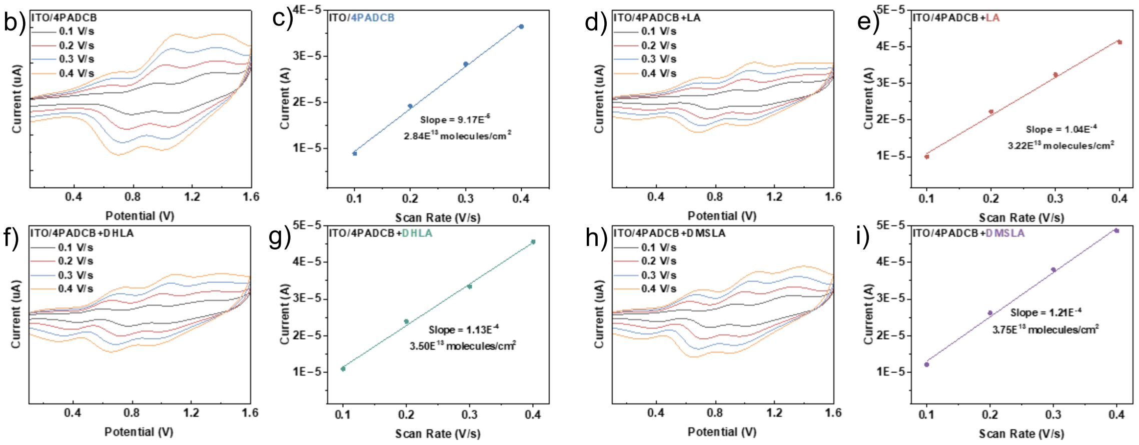

Supplementary Figure 16: a) Table presenting the calculated coating efficiencies of SAM and its mixed form with LA analogues on the ITO electrode, b-c) cyclic voltammograms and current-voltage (I–V) characteristics of ITO modified with SAM (4PADCB) alone, d-e) with SAM+LA, f-g) with SAM+DHLA, and h-i) with SAM+DMSLA.

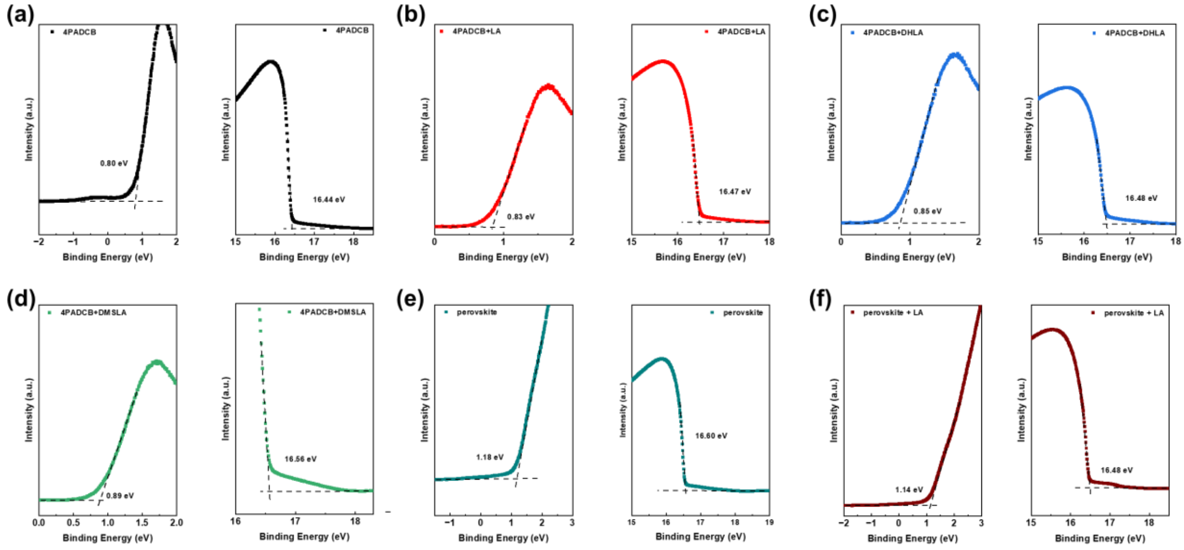

Supplementary Figure 17: **a)** UPS spectra of a) 4PADCB, b) 4PADCB+LA, c) 4PADCB+DHLA, d) 4PADCB+DMSLA, e) perovskite, and f) perovskite+LA films deposited on ITO substrate, respectively.

Supplementary Table 1: Electronic parameters of SAM (control), its modified forms, perovskite films w/wo LA from the UPS spectra.

Sample	E_{cutoff}(eV)	E_{onset}(eV)	VBM(eV)	Work function(eV)
4PADCB	16.44	0.80	-5.47	-4.76
4PADCB + LA	16.47	0.83	-5.56	-4.73
4PADCB + DHLA	16.48	0.85	-5.57	-4.72
4PADCB + DMSLA	16.56	0.89	-5.53	-4.64
Perovskite	16.6	1.18	-5.78	-4.6
Perovskite +LA	16.48	1.14	-5.86	-4.72

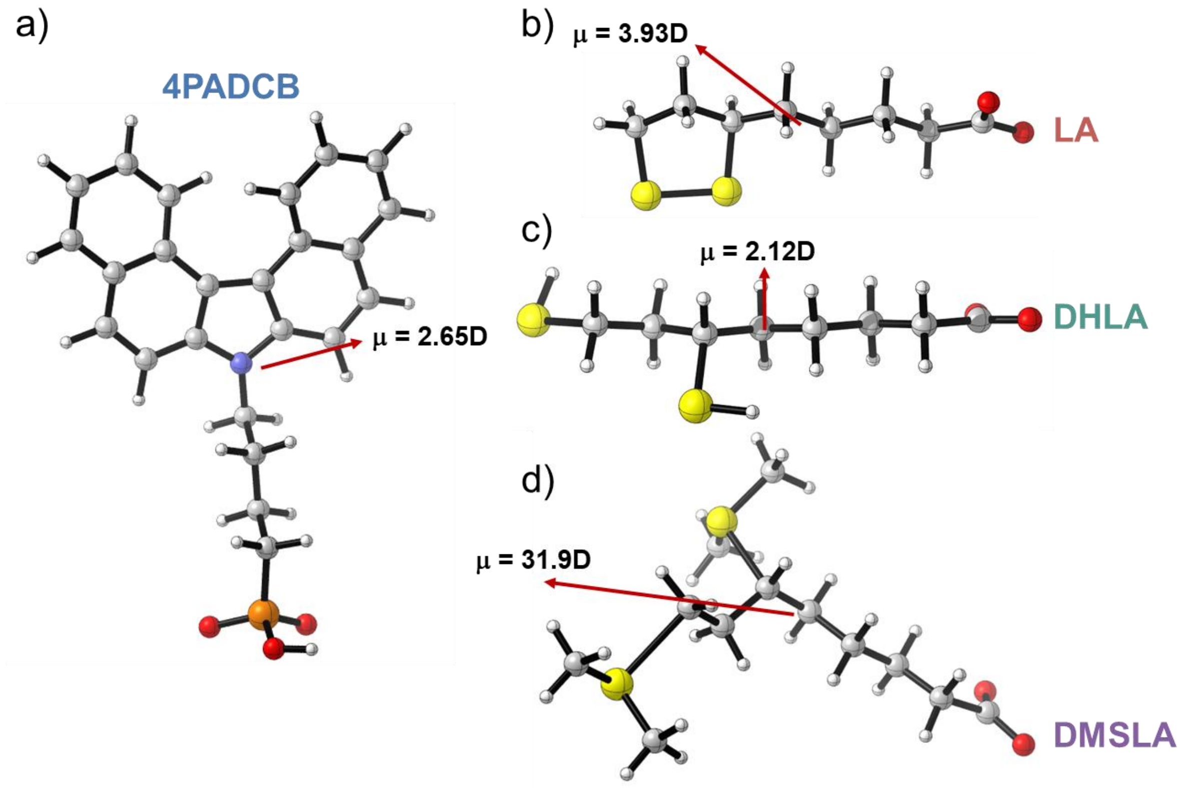

Supplementary Figure 18: Optimized molecular structures and calculated dipole moments of the surface modifiers used in this work: a) 4PADCB, b) LA, c) DHLA, and d) DMSLA. Dipole moments were computed using density functional theory (DFT) at the B3LYP/6-31G(d) level in the gas phase. The vectors indicate the direction and magnitude of the dipole moment, which influences the interfacial vacuum level alignment when assembled on ITO substrates.

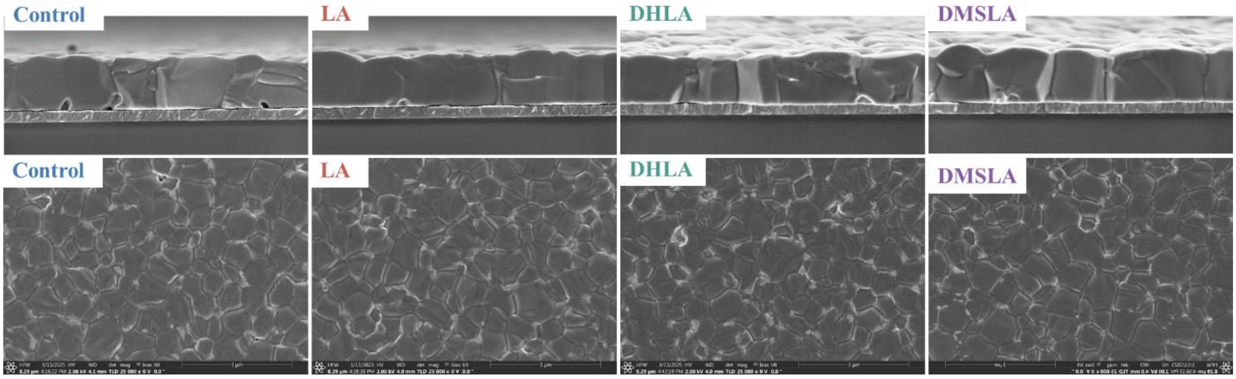

Supplementary Figure 19: SEM cross-section and top-view images of perovskite films. The control group corresponds to perovskite films without any LA or its derivatives, whereas the other samples include LA and its derivatives at the perovskite–HTL interface, along with a fixed amount of LA incorporated within the bulk structure.

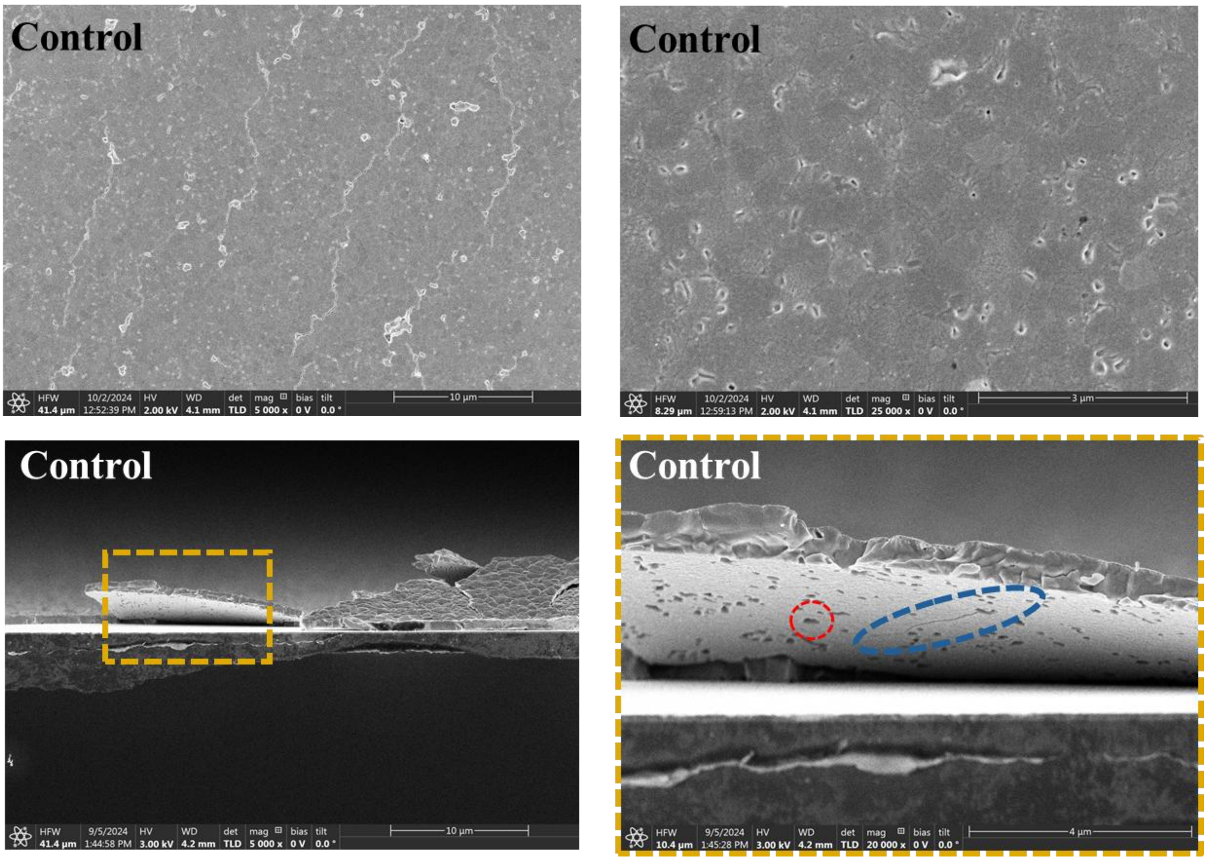

Supplementary Figure 20: SEM images from the bottom face and the cross-section of the Control film. Nano-level voids (red dashed circle) and cracks (blue dashed ellipse) were observed at the interface that contacted with the SAM in the film Control.

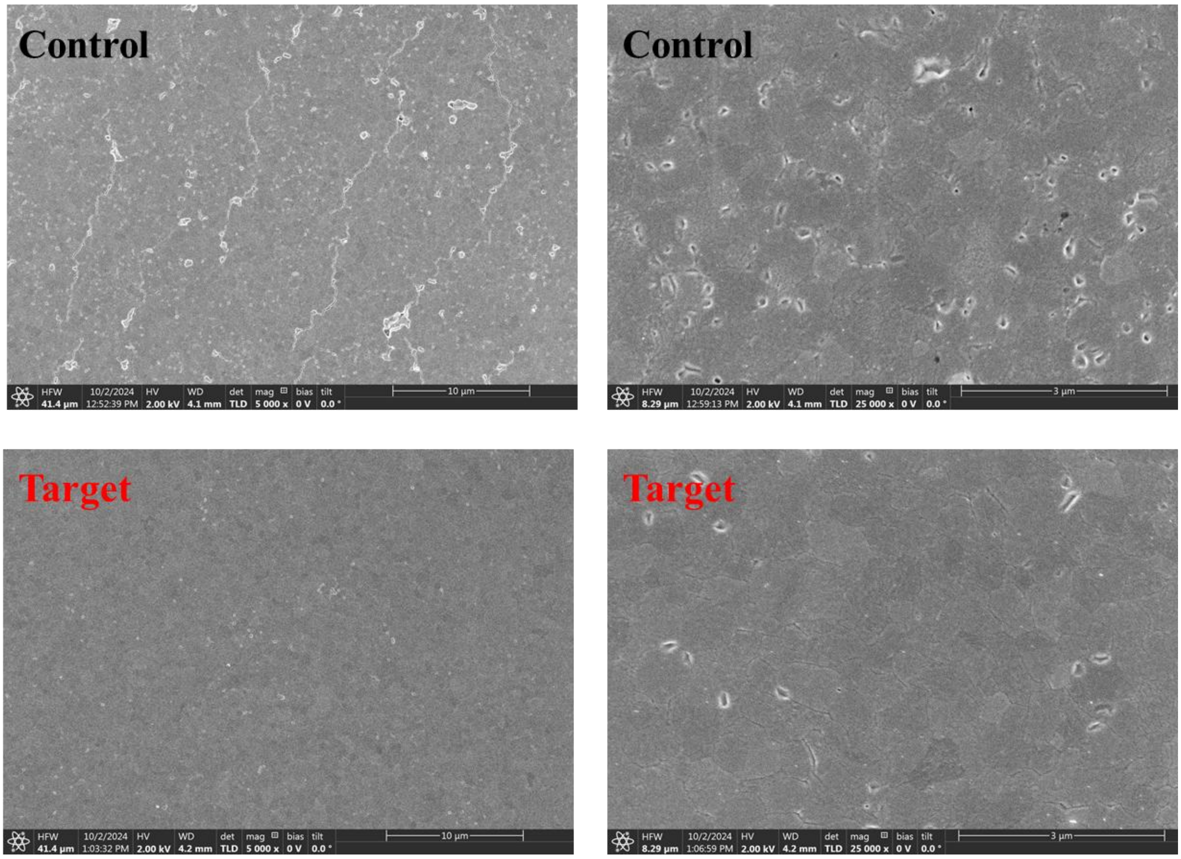

Supplementary Figure 21: SEM images from the bottom face of the Control and Target films. The voids observed in the **Control** at the nano level were almost eliminated in films treated with LA (Target) and its derivatives.

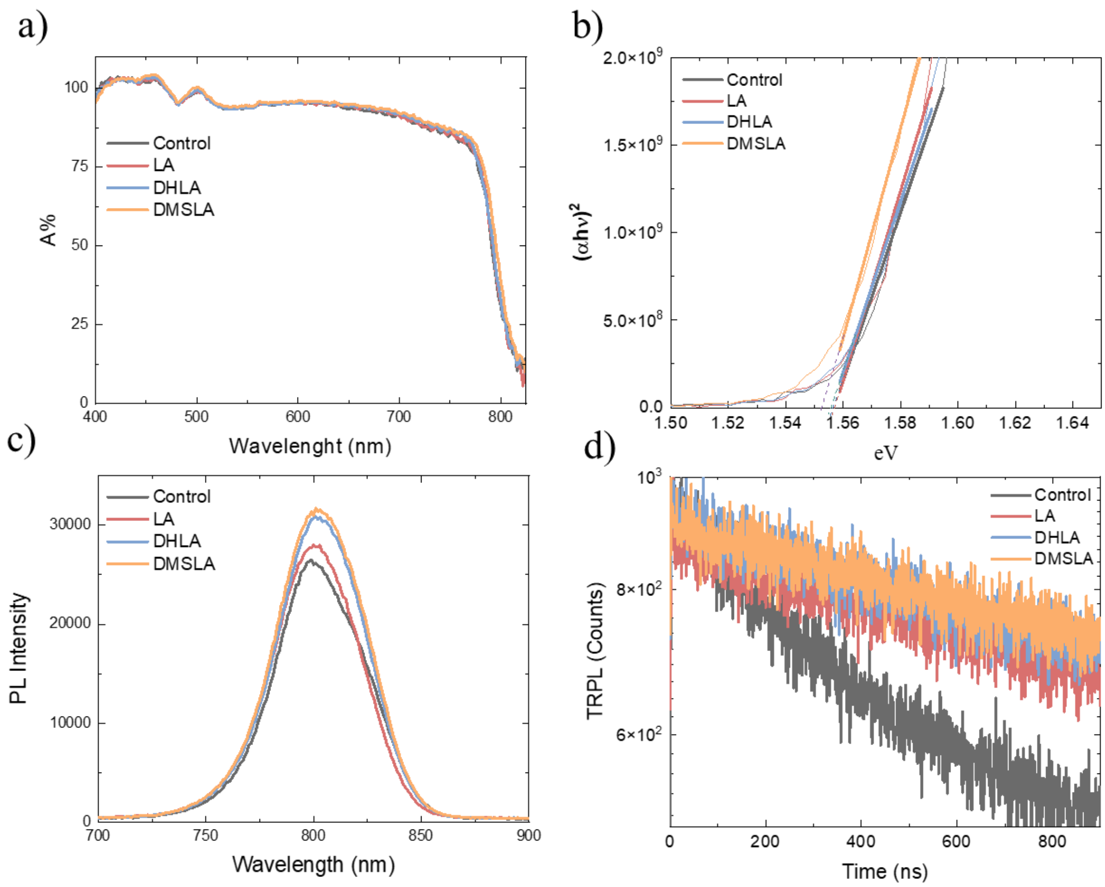

Supplementary Figure 22: a) UV–vis absorption spectrum, b) Tauc plots, c) Steady-state PL spectra (excitation wavelength of 375 nm), d) Time-resolved PL decay curves of control and target films. Based on the constructed Tauc plots, the estimated optical band gaps for the control and target films are as follows: Control – 1.556 eV, LA – 1.556 eV, DHLA – 1.555 eV, and DMSLA – 1.553 eV.

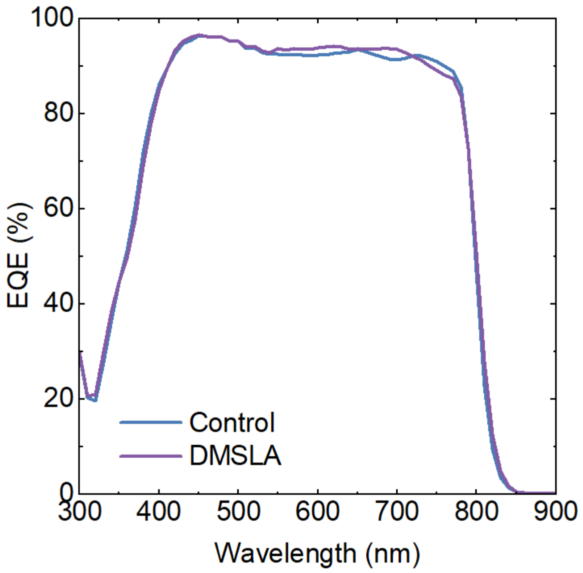

Supplementary Figure 23: EQE results of control (without LA and its derivatives) and DMSLA-incorporated solar cell devices.

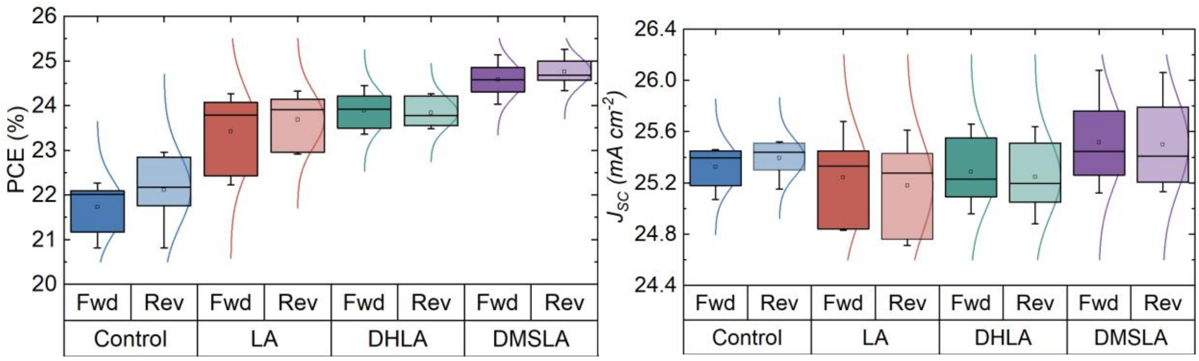

Supplementary Figure 24: Statistical distribution of a) PCE (%), and b) *J*sc values for the solar cell devices fabricated at LMU Munich. In the combined box–violin plots, the center line represents the median; the box limits correspond to the 25th and 75th percentiles; whiskers extend from the minimum to the maximum values; squares denote the mean; and the overlaid curves indicate the data distribution density. Error bars represent standard deviation.

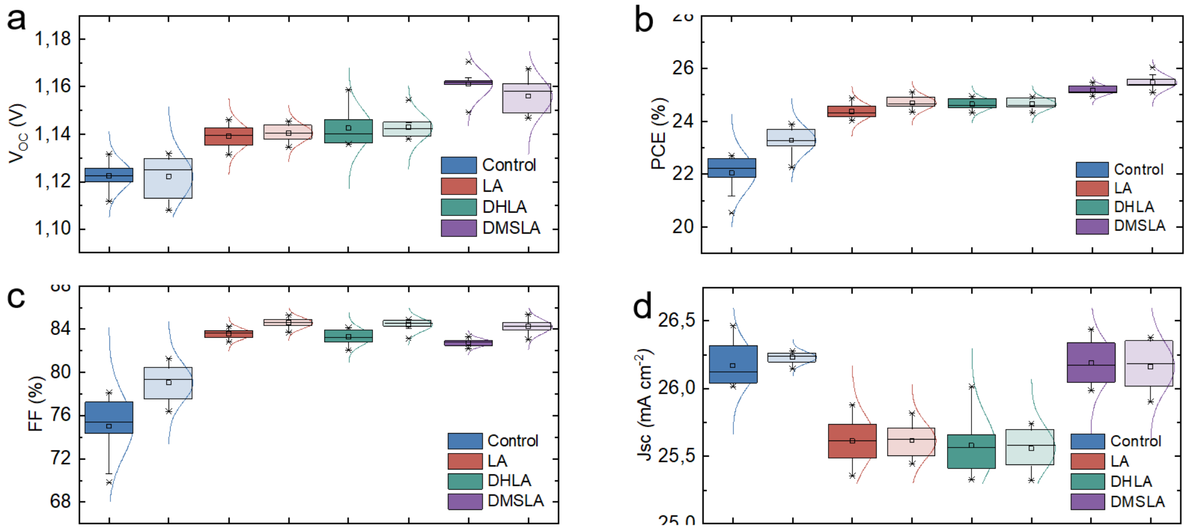

Supplementary Figure 25: Statistical distribution of a) V_{OC}, b) PCE (%), c) FF (%), and d) J_{SC} values for the solar cell devices fabricated at Tianjin University. Darker-coloured data points correspond to the forward *J–V* scans, while lighter-coloured data points correspond to the reverse *J–V* scans. In the combined box–violin plots, the center line represents the median; the box limits correspond to the 25th and 75th percentiles; whiskers extend from the minimum to the maximum values; squares denote the mean; and the overlaid curves indicate the data distribution density. Error bars represent standard deviation.

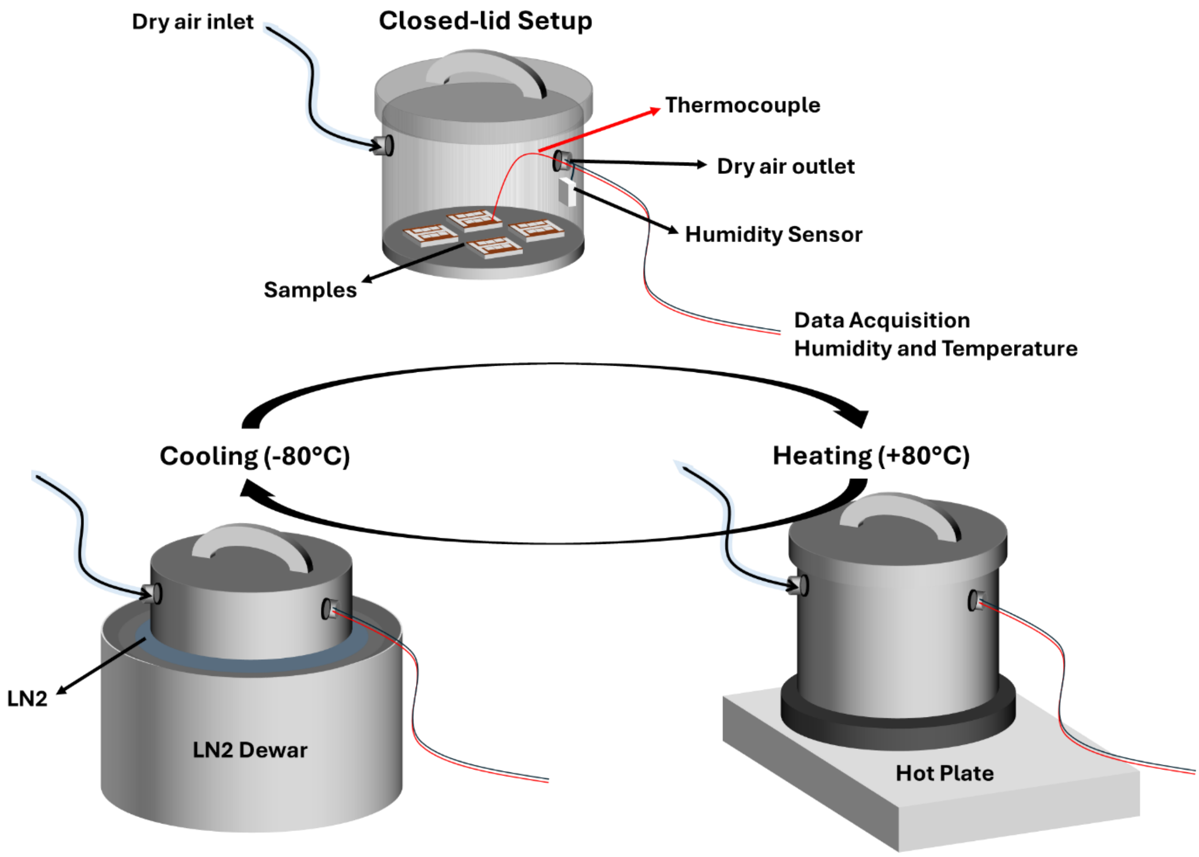

Supplementary Figure 26: Schematic representation of the thermal fatigue experimental setup.

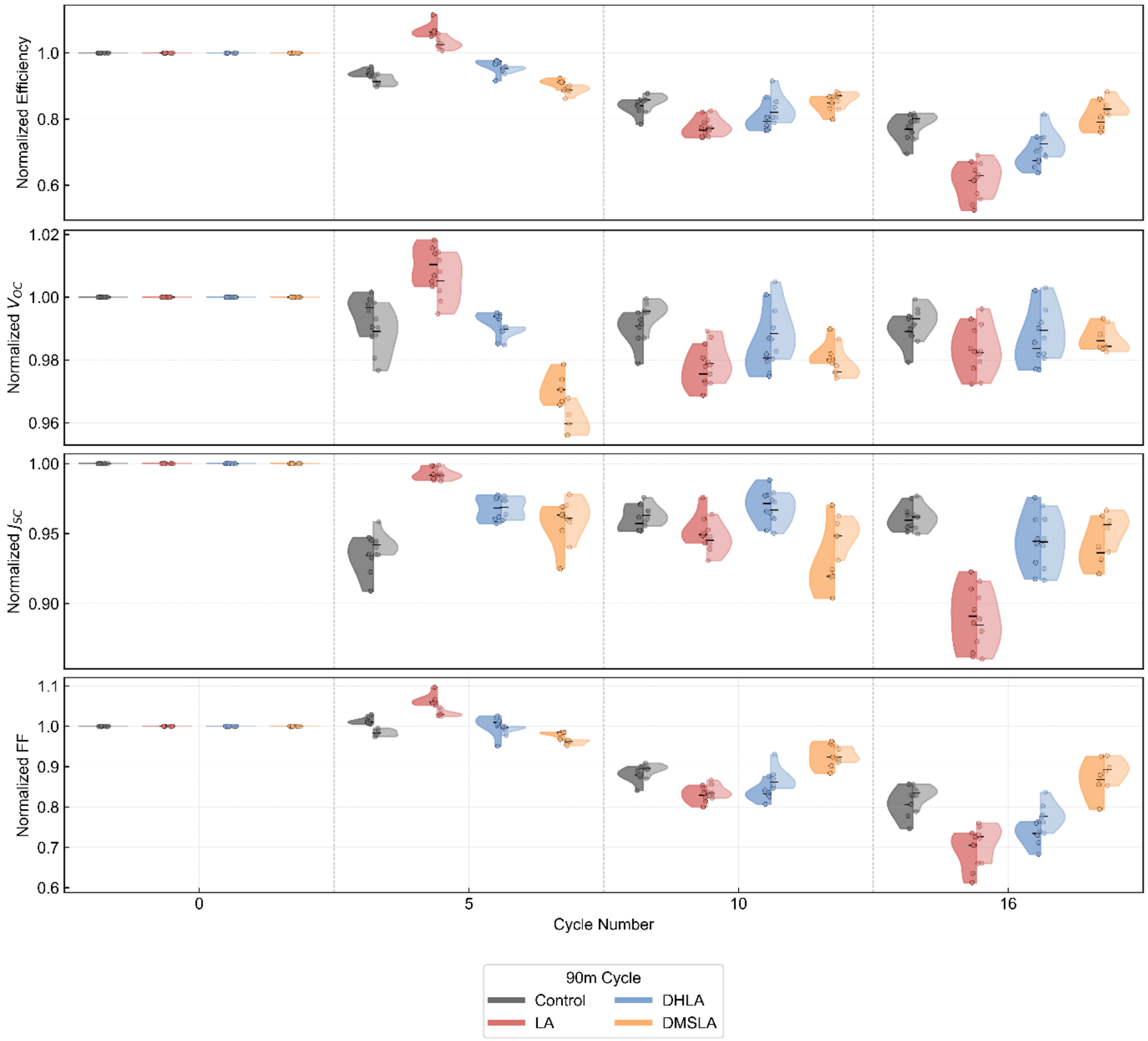

Supplementary Figure 27. Statistical distribution of photovoltaic parameters under 90-minute thermal-cycling conditions for devices fabricated at LMU Munich. Boxplots show the normalized (a) PCE, (b) V_{OC}, (c) J_{SC}, and (d) FF for control, LA-treated, DHLA-treated, and DMSLA-treated perovskite solar cells measured after encapsulation (baseline), and after 1, 3, 8, and 16 thermal cycles. Each data point corresponds to an individual device pixel (six pixels per device). Parameter values are normalized to those measured at cycle 0 for each device. Darker-coloured data points correspond to the forward *J–V* scans, while lighter-coloured data points correspond to the reverse *J–V* scans.

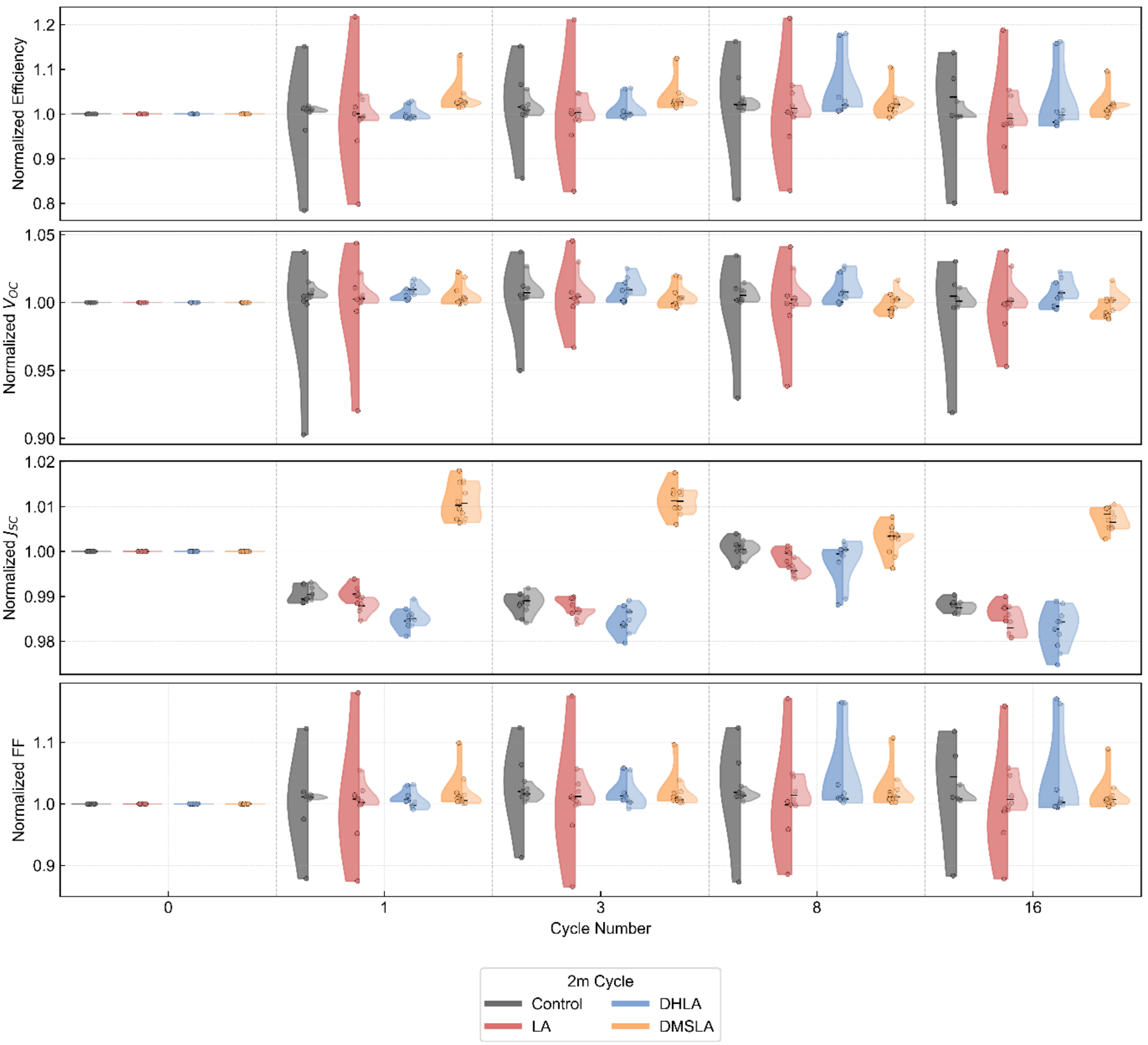

Supplementary Figure 28. Statistical distribution of photovoltaic parameters under 2-minute rapid thermal-cycling conditions for devices fabricated at LMU Munich. Boxplots show the normalized (a) PCE, (b) V_{OC}, (c) J_{SC}, and (d) FF for control, LA-treated, DHLA-treated, and DMSLA-treated devices evaluated after 0, 1, 3, 8, and 16 cycles. Each point represents a single active pixel on the device. All photovoltaic parameters remain essentially unchanged throughout cycling, confirming that rapid temperature transitions with short exposure times impose negligible stress and further supporting that performance degradation is governed by cumulative thermal-exposure duration rather than the number of cycles. Darker-coloured data points correspond to the forward *J*–*V* scans, while lighter-coloured data points correspond to the reverse *J*–*V* scans.

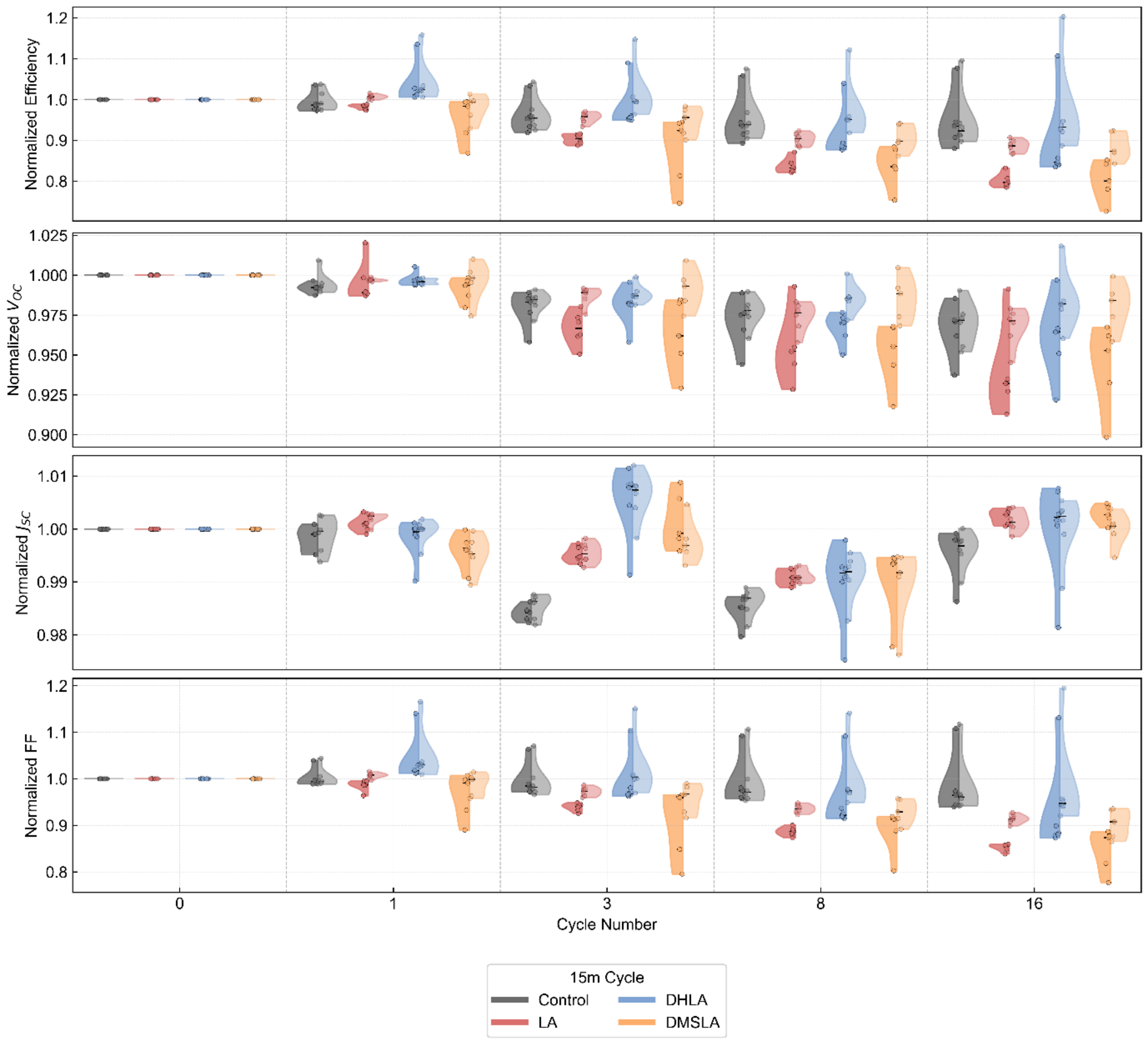

Supplementary Figure 29. Statistical distribution of photovoltaic parameters under 15-minute thermal-cycling conditions for devices fabricated at LMU Munich. Boxplots show the normalized (a) PCE, (b) V_{OC}, (c) J_{SC}, and (d) FF for control, LA-treated, DHLA-treated, and DMSLA-treated solar cells measured after 0, 1, 3, 8, and 16 cycles. Each data point corresponds to an individual device pixel (six pixels per device). Values are normalized to the corresponding parameter measured at cycle 0 for each sample. All device groups indicate moderate thermomechanical degradation under moderate cycle duration, demonstrating that cycle count alone is not a sufficient predictor of fatigue when compared to 90-minute thermal-cycling conditions. Darker-coloured data points correspond to the forward *J*–*V* scans, while lighter-coloured data points correspond to the reverse *J*–*V* scans.

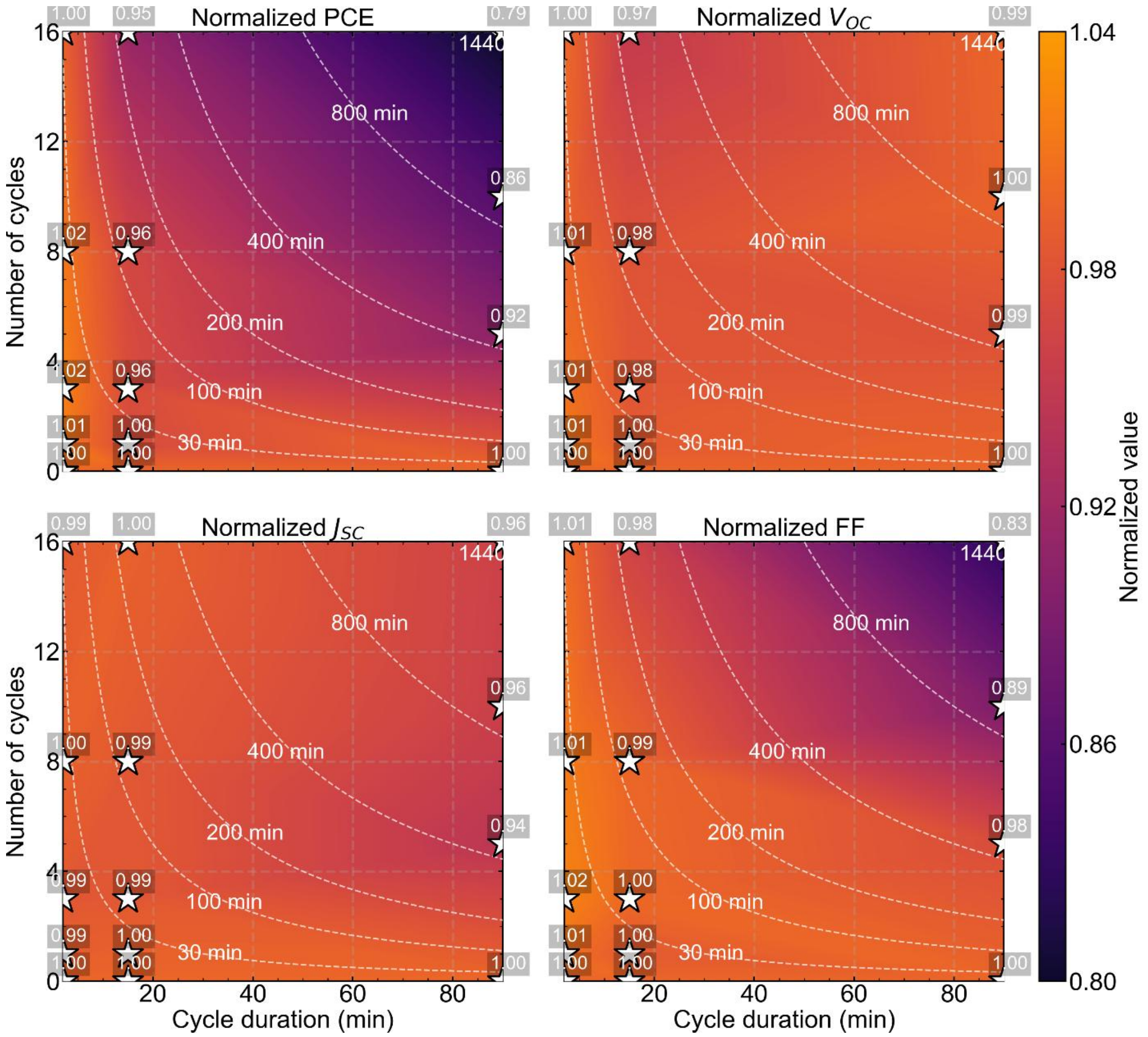

Supplementary Figure 30. Contour maps showing the dependence of normalized photovoltaic parameters on cycle duration and number of thermal cycles for control perovskite solar cells. Panels display the normalized (a) PCE, (b) V_{OC}, (c) J_{SC}, and (d) FF across a matrix of cycle durations (2–90 minutes) and cycle counts (0–16). Star symbols indicate the experimentally tested conditions in this work (2-min, 15-min, and 90-min cycling, each for 16 cycles). White dashed lines represent contours of constant total thermal-exposure time. All parameters are normalized to their respective values at cycle 0. Contour maps are generated by interpolation of discrete measured data points. These plots reveal that degradation aligns more strongly with total thermal-exposure time rather than cycle count, with negligible changes observed for short-duration cycles and measurable performance drops appearing only for extended exposure (e.g., 90-min cycles).

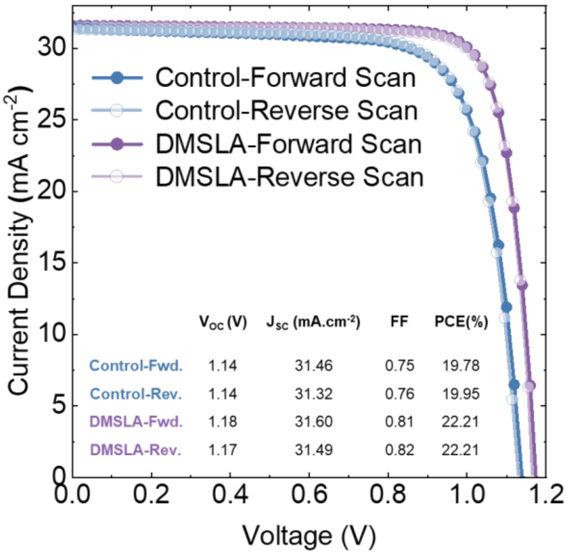

Supplementary Figure 31. *J–V* curves and the corresponding device parameters of the devices at 1360 W m^{-2} (AM0 approximation).

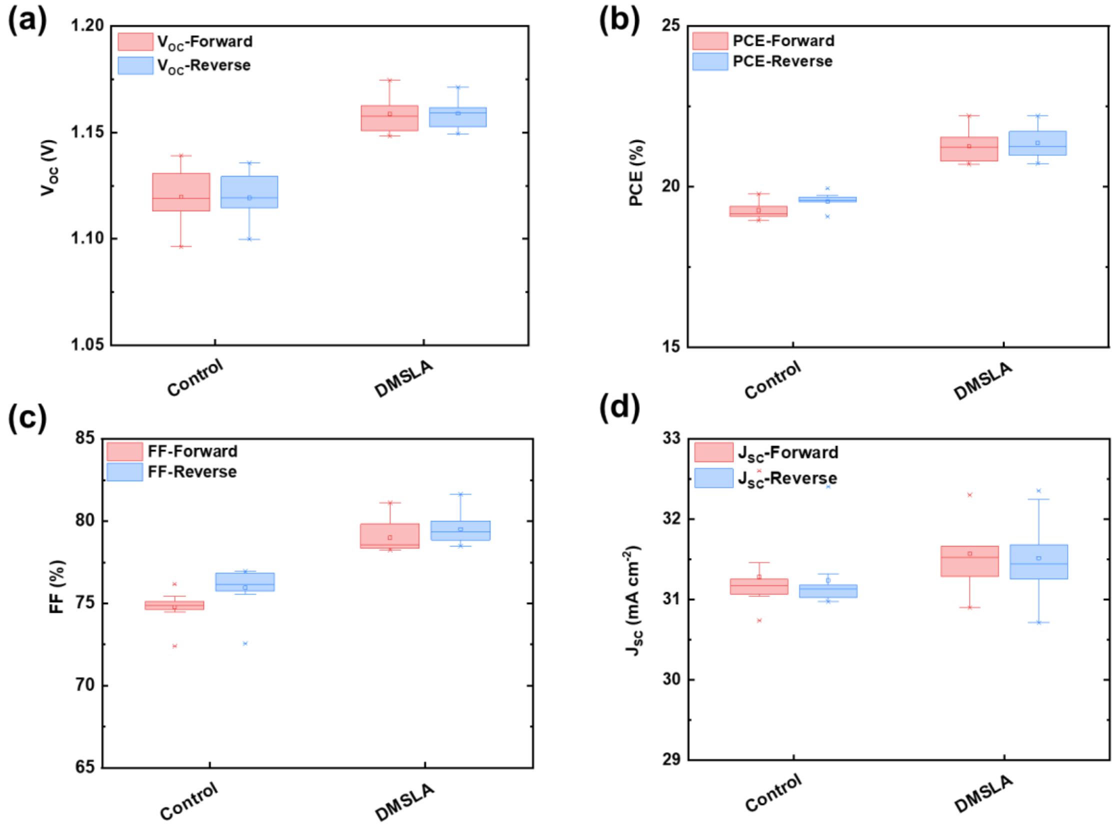

Supplementary Figure 32. Statistical distribution of a) V_{OC}, b) PCE, c) FF, and (d) J_{SC} values of the devices at 1360 W m^{-2}. Each condition had 10 devices. In the box plots, the center line represents the median; the box limits correspond to the 25th and 75th percentiles; whiskers

extend from the minimum to the maximum values; squares denote the mean; and error bars represent standard deviation.

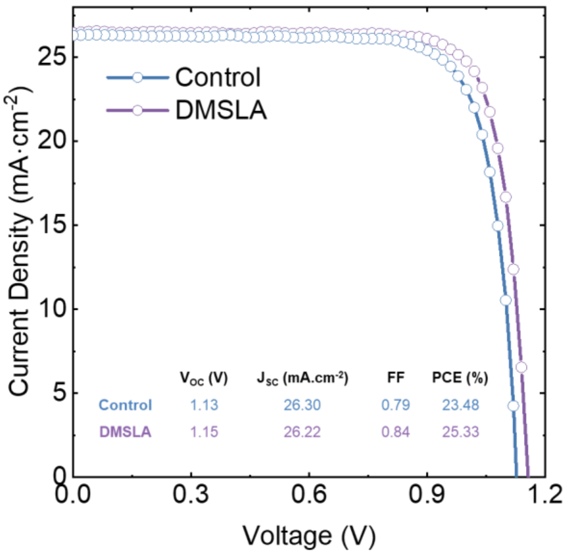

Supplementary Figure 33. The corresponding *J–V* curves and the initial PCE data for the MPPT.

Supplementary Table 2. Summary of reported thermal-cycling studies on perovskite solar cells under space-relevant or terrestrial conditions.

Reference	Target Application	Temp Range (°C)	Cycles	Cycle Duration	Initial PCE (%)	Final PCE (%)	PCE Retention	Encapsulation	Key Finding
2	General space	-123 to +157	5	300 m	24.39	20.15	82.7%	Ultrahigh vacuum; no encapsulation	Absorber decomposition to Pb/PbI2, volatile organics, ion migration under vacuum thermal cycling
3	General space	-160 to +150	50	30 °C min^{-1} rate, 20 m	Not reported	Not reported	97% (AM0)	Not specified	Temperature-dependent phase transitions and recoverable lattice strain; widest temperature range tested
4	Near-space/cryogenic	-143 to +17	120	81 m	24.34 (at 150K)	~17.5 (estimated)	72%	Not specified	PAN additive stabilized lattice at low temperatures; 72% retention after 120 cryogenic cycles (highest cycle count at extreme T)
5	Terrestrial	-60 to +80	120	18 m	24.6	Not reported	93.9% at 80°C and 88.7% at −60°C	Unencapsulated	Impact of the ordered dipolar structure on the operational stability
6	General space	-40 to +80	200		25.78 (rigid), 24.54 (flexible)	Not stated	>95%	Unencapsulated	In-situ cross-linked polymer; 200 thermal cycles + 10,000 bending cycles; >95% retention
7	LEO CubeSat	-40 to +80	250	25 m	24.28	9.7 (estimated)	40%	UV cut film and polyimide/Kapton tape/ UV light curable glue/1 mm glass	HTM comparison under simulated space environment; carbon HTM outperformed organic/inorganic HTMs
8	Terrestrial/space readiness	-40 to +85	5000	5 m	21.3	17.2	81%	Unencapsulated	Alkyl-ammonium additive reduced residual stress; highest cycle count for perovskites (2,500 cycles)
9	Extreme temperature cycling	-80 to +80	200	40 m	Not stated	Not stated	>90%	Vacuum-packed	Non-volatile 4CP stabilizes Li^+, suppresses oxides, enabling ~26% efficient, shock-stable perovskite cells
This work	Extreme temperature cycling	-80 to +80	16	90 m			84%	Space grade encapsulation, DOWSIL 93-500/0.2 mm glass	Dual grain-boundary and interfacial reinforcement improves resistance to thermomechanical degradation under repeated extreme temperature cycling.

Supplementary References

1 Szostak, R. *et al.* Nanoscale mapping of chemical composition in organic-inorganic hybrid perovskite films. *Science advances* **5**, eaaw6619 (2019).

2 Lu, X. *et al.* In-Situ Investigation of Perovskite Solar Cells Under Coupled Vacuum-Thermal Cycling in Extreme Space Environments. (2025).

3 Li, G. *et al.* Structure and performance evolution of perovskite solar cells under extreme temperatures. *Advanced Energy Materials* **12**, 2202887 (2022).

4 Yang, Z., Wu, Y., Yang, N., Yang, R. & Hao, Y. Revealing the role of polyacrylonitrile for highly efficient and stable perovskite solar cells at extremely low temperatures. *Advanced Energy Materials* **14**, 2400638 (2024).

5 Li, G. *et al.* Highly efficient pin perovskite solar cells that endure temperature variations. *Science* **379**, 399-403 (2023).

6 Li, Z. *et al.* In-Situ Cross-Linked Polymers for Enhanced Thermal Cycling Stability in Flexible Perovskite Solar Cells. *Angewandte Chemie International Edition* **64**, e202421063 (2025).

7 Bautista, I. Z. C. *et al.* Perovskite Solar Cells in Space: Evaluation of Perovskite Solar Cell Hole Transport Material in Space Environment. *Transactions of the Japan Society for Aeronautical and Space Sciences* **65**, 95-107 (2022).

8 Chen, M. *et al.* Stress Engineering for Mitigating Thermal Cycling Fatigue in Perovskite Photovoltaics. *ACS Energy Letters* **9**, 2582-2589 (2024). https://doi.org/10.1021/acsenergylett.4c00988

9 Kim, K. *et al.* Non-volatile solid-state 4-(N-carbazolyl)pyridine additive for perovskite solar cells with improved thermal and operational stability. *Nature Energy* (2025). https://doi.org/10.1038/s41560-025-01864-z
