# Supplementary material for: Perovskite solar cells with enhanced thermal fatigue resistance under extreme temperature cycling
Source: Nat Commun. 2026 Mar 9;17:3669. doi: 10.1038/s41467-026-70293-7 (PMC13100044; doi:10.1038/s41467-026-70293-7)
Supplement: Supplementary file 2 — Reporting Summary [file 41467_2026_70293_MOESM2_ESM.pdf]

Corresponding author(s): Fei Zhang
Erkan Aydin

Solar Cells Reporting Summary

Nature Research wishes to improve the reproducibility of the work that we publish. This form is intended for publication with all accepted papers reporting the characterization of photovoltaic devices and provides structure for consistency and transparency in reporting. Some list items might not apply to an individual manuscript, but all fields must be completed for clarity.

For further information on Nature Research policies, including our data availability policy, see Authors & Referees.

ü Experimental design

Please check: are the following details reported in the manuscript?

1. Dimensions

Area of the tested solar cells	[x] Yes	For single junction cell, the area (0.1 cm2).
	[ ] No	*Explain why this information is not reported/not relevant.*
Method used to determine the device area	[x] Yes	For in-house measurement, shadow mask is used to determine the active area.
	[ ] No	*Explain why this information is not reported/not relevant.*

2. Current-voltage characterization

Current density-voltage (J-V) plots in both forward and backward direction	[x] Yes	Given in Figure 3b (from LMU Munich), and Figure 3c (from Tianjin University).
	[ ] No	*Explain why this information is not reported/not relevant.*
Voltage scan conditions<br>*For instance: scan direction, speed, dwell times*	[x] Yes	Given in solar cell characterization section in Methods (supplementary).
	[ ] No	*Explain why this information is not reported/not relevant.*
Test environment<br>*For instance: characterization temperature, in air or in glove box*	[x] Yes	Given in solar cell characterization section in Methods (supplementary).
	[ ] No	*Explain why this information is not reported/not relevant.*
Protocol for preconditioning of the device before its characterization	[ ] Yes	*State where this information can be found in the text.*
	[x] No	*Explain why this information is not reported/not relevant.*
Stability of the J-V characteristic<br>*Verified with time evolution of the maximum power point or with the photocurrent at maximum power point; see ref. 7 for details.*	[x] Yes	Given in Figure 4d, and Figure 3c.
	[ ] No	

3. Hysteresis or any other unusual behaviour

Description of the unusual behaviour observed during the characterization	[ ] Yes	*State where this information can be found in the text.*
	[x] No	*Explain why this information is not reported/not relevant.*
Related experimental data	[x] Yes	The negligible hysteresis was observed in J-V plots for the reverse and forwards scans.
	[ ] No	*Explain why this information is not reported/not relevant.*

4. Efficiency

External quantum efficiency (EQE) or incident photons to current efficiency (IPCE)	[x] Yes	Given in Figure S19 (supplementary).
	[ ] No	*Explain why this information is not reported/not relevant.*
A comparison between the integrated response under the standard reference spectrum and the response measure under the simulator	[ ] Yes	*State where this information can be found in the text.*
	[x] No	*Explain why this information is not reported/not relevant.*
For tandem solar cells, the bias illumination and bias voltage used for each subcell	[ ] Yes	*Explain why this information is not reported/not relevant.*
	[x] No	

5. Calibration

Light source and reference cell or sensor used for the characterization	[x] Yes	A Newport Oriel Sol 2A solar simulator was used for in-house measurements, and the intensity was corrected using a Fraunhofer ISE-calibrated reference cell, along with a spectrophotometric correction against the NREL AM 1.5G reference spectrum.
	[ ] No	*Explain why this information is not reported/not relevant.*

Confirmation that the reference cell was calibrated and certified

- [x] Yes — Fraunhofer ISE-certified silicon reference cell (Serial number: 032-2011).
- [ ] No — *Explain why this information is not reported/not relevant.*

Calculation of spectral mismatch between the reference cell and the devices under test

- [ ] Yes — *State where this information can be found in the text.*
- [x] No — *Explain why this information is not reported/not relevant.*

6. Mask/aperture

Size of the mask/aperture used during testing

- [x] Yes — Given in solar cell characterization section Methods.
- [ ] No — *Explain why this information is not reported/not relevant.*

Variation of the measured short-circuit current density with the mask/aperture area

- [ ] Yes — *State where this information can be found in the text.*
- [x] No — We report our results always with aperture. Edge effects are minimized. Fraunhofer ISE CalLab measured the aperture area by themselves.

7. Performance certification

Identity of the independent certification laboratory that confirmed the photovoltaic performance

- [x] Yes — Results were cross-validated (LMU-Tianjin) at an independent laboratory.
- [ ] No — *Explain why this information is not reported/not relevant.*

A copy of any certificate(s)
Provide in Supplementary Information

- [ ] Yes — *Explain why this information is not reported/not relevant.*
- [x] No

8. Statistics

Number of solar cells tested

- [x] Yes — For different conditions, different number of devices were fabricated. For single junction cell > 6 device fabricated for each condition to show the reproducibility.
- [ ] No — *Explain why this information is not reported/not relevant.*

Statistical analysis of the device performance

- [x] Yes — Given in Figure 3f, Figure S20, and Figure S21(supplementary).
- [ ] No — *Explain why this information is not reported/not relevant.*

9. Long-term stability analysis

Type of analysis, bias conditions and environmental conditions
For instance: illumination type, temperature, atmosphere humidity, encapsulation method, preconditioning temperature

- [x] Yes — Given in Figure 4a (Thermal fatigue test), and Figure 4d (MPPT).
- [ ] No
